# Initial investigation of molecular phenotypes of airway mast cells and cytokine profiles in equine asthma

**DOI:** 10.3389/fvets.2022.997139

**Published:** 2023-01-11

**Authors:** Jane S. Woodrow, Melissa Hines, Carla Sommardahl, Bente Flatland, Yancy Lo, Zhiping Wang, Mary Katie Sheats, Elizabeth M. Lennon

**Affiliations:** ^1^Department of Comparative and Experimental Medicine, College of Veterinary Medicine, University of Tennessee, Knoxville, Knoxville, TN, United States; ^2^Department of Clinical Sciences and Advanced Medicine, College of Veterinary Medicine, University of Pennsylvania, Philadelphia, PA, United States; ^3^Department of Large Animal Clinical Sciences, College of Veterinary Medicine, University of Tennessee, Knoxville, Knoxville, TN, United States; ^4^Department of Biomedical and Diagnostic Sciences, College of Veterinary Medicine, University of Tennessee, Knoxville, Knoxville, TN, United States; ^5^Bioinformatics Core, Institute for Biomedical Informatics, University of Pennsylvania, Philadelphia, PA, United States; ^6^Department of Clinical Sciences, Comparative Medicine Institute, College of Veterinary Medicine, North Carolina State University, Raleigh, NC, United States

**Keywords:** equine, asthma, mast cell, cytokine, chemokine, airway

## Abstract

Equine asthma is a naturally occurring lung disease characterized by chronic, partially reversible airway obstruction, pulmonary remodeling, and lower airway inflammation. Asthma is currently divided into two major groups, mild to moderate asthma (mEA) and severe asthma (sEA), but further subtyping by phenotype (i.e., clinical presentation) and/or endotype (i.e., cellular mechanisms) may be warranted. For this study, we were interested in further investigation of cellular and inflammatory characteristics of EA, including airway mast cells. The purpose of this study was to: (1) compare mast cell protease mRNA expression between healthy and asthmatic horses, (2) analyze the cytokine profile present in BALF of currently defined equine asthma groups, and (3) use these data to evaluate potential biomarkers of defined asthma groups. We hypothesized that there would be significant differences in the cellular mast cell phenotypes (i.e., mucosal vs. connective tissue) and cytokine profiles in the BALF of asthmatic vs. healthy horses and across asthma groups. We assert these characteristics may inform additional subtypes of equine asthma. Adult horses were recruited from the institution's teaching herd and clinical caseload. Mast cell protease gene expression of the BALF cellular component and multiplex bead immunoassay for cytokine concentrations in the BALF supernatant were investigated. Airway mast cells primarily expressed tryptase, with low levels of chymase. No significant changes in protease expression were detected across groups. Horses with severe asthma had increased TNF-α, CXCL-8, and IFN-γ concentrations in BALF supernatant. Multidimensional analysis demonstrated healthy and mEA horses have overlapping characteristics, with sEA separating from the other groups. This difference was primarily due to BALF neutrophil and lymphocyte concentrations. These study results further inform understanding of EA immunopathology, and future studies designed to investigate asthma phenotypes and endotypes. Ultimately, a better understanding of these groups could help identify novel therapeutic strategies.

## 1. Introduction

Equine asthma is a chronic inflammatory lung disease characterized by enhanced bronchial reactivity; chronic, partially reversible airflow obstruction; pulmonary remodeling, including fibrosis; and lower airway inflammation (1–3). Equine asthma prevalence in the Northern hemisphere is about 14%, and the disease can be not only frustrating to manage, but even career or life ending for the horse (4, 5). A 2016 consensus statement by the American College of Veterinary Medicine defined two categories of equine asthma syndrome (EAS), currently designated as mild to moderate asthma (mEA) and severe asthma (sEA) (1). The diagnosis of asthma is based on history, physical examination, bronchoalveolar lavage fluid (BALF) cytology, +/– lung function tests. BALF cytological findings supportive of asthma include an increased percentage of neutrophils (most pronounced in severe asthma), mast cells, eosinophils, or some combination of increase in these cells without evidence of bacterial or fungal pneumonia. In human medicine, a diagnosis of asthma can be classified by phenotype (typical clinical presentation and/or triggers), endotype (defined by molecular pathways), genetic variation, or treatment response.

In both horses and humans, asthma immune responses have been broadly characterized by a Type 2 immune response, termed Type 2 high; and those with a mixed immune response, termed Type 2 low or non-type 2 (6, 7). In human asthma, type 2 high is most associated with increases in airway IL-4, IL-5, and IL-13 as drivers of eosinophilic inflammation and type 2 low, which has been less well studied, is recognized as the absence of type 2 cytokines and non-eosinophilic inflammation. In studies of asthmatic horses, these endotypes have been identified in both EA phenotypes, mEA and sEA, suggesting classification systems other than mEA and sEA may be needed to describe underlying disease immunopathology (8). The cytokine profiles of mEA and sEA, either at the protein or mRNA level, may support a type 1, type 2, type 17, or mixed immune response, depending on the study (9–21). Inconsistencies in reported cytokine profiles and subcategorizing EA based on clinical severity rather than immune characteristics of the disease have limited ability to fully describe the pathogenesis of EA and establish clinically relevant disease categories, which ultimately limits diagnostic, treatment, and management abilities. Understanding how immune cells are activated and recruited to continue the inflammatory cycle is key to curtailing inflammation. For example, horses with severe asthma have a markedly increased BALF neutrophil proportions. This is presumptively the result of increased CXCL8 gene expression (a chemokine involved in neutrophil recruitment), as has been shown in one small clinical study (22). Further defining the cytokine and chemokine milieus in asthmatic horses and how these may vary between types of asthma is key to identifying targets for therapy and improving tailored therapy for individual horses.

In human asthma, endotype classification (Type 2 high vs. Type 2 low) is further defined by the predominant inflammatory cells present in the airways, such as eosinophilic, neutrophilic, or paucigranulocytic (23–25). The discrimination of asthma endotype in humans is essential to understanding pathogenesis, but is also clinically relevant since different endotypes respond to different treatments (26, 27). Discrimination of asthma endotype based on cytokine profiles is essential to informing specific biologic therapy with drugs such as omalizumab, which inhibits binding of IgE to the receptor on mast cells and basophils (28, 29). Studies using “omic” technologies have implicated innate immune cells, such as basophils and mast cells, in the pathogenesis of inflammation (30, 31). The presence of mast cells in human asthmatics' BALF and induced sputum samples has been shown to correlate with asthma phenotype, including response to therapy (32–35).

Mast cells are classically identified by expression of CD117 (c-kit), the receptor for stem cell factor (SCF), and FcεRI, the high affinity receptor for IgE. In humans, two main phenotypes of mast cells are recognized based on the proteases they contain: tryptase-only containing mast cells (MC_T_, mucosal location); and tryptase, chymase, and carboxypeptidase A3 containing mast cells (MC_TC_, connective tissue location) (36, 37). Healthy human lungs predominantly contain mast cells of the mucosal type (MC_T_), and asthma is associated with an increase in MC_T_ (predominantly mild asthma) and increasing chymase expression (severe asthma) (32, 38). Cytological identification of mast cells in induced human sputum is difficult due to low cell prevalence and release of cytoplasmic protease content by mast cells which precludes mast cell identification by staining for cytoplasmic proteases. Therefore, using protease mRNA expression, termed molecular phenotyping, is preferred to identify mast cells. Using molecular phenotyping of human asthma samples, a third mast cell type (tryptase^+^/carboxypeptidase A3^+^/chymase^low^) was identified that correlated with eosinophilic asthma and increased responsiveness to corticosteroids (33, 34). Given their clinical relevance in human asthma, phenotypes of lung mast cells in EA warrant further investigation (39, 40).

The objectives of this study were to: (1) compare mast cell protease mRNA expression between healthy and asthmatic horses, (2) analyze the cytokine profile present in BALF of currently defined equine asthma groups, and (3) use these data to evaluate potential biomarkers of defined asthma groups. We hypothesized that healthy and asthmatic horses would have significantly different BALF mast cell protease mRNA expression and cytokine/chemokine levels that would correlate with BALF cytology results and health status.

## 2. Material and methods

### 2.1. Study population

Horses were enrolled from the institution's equine hospital caseload and research herd from February 2018 to April 2019. Client-owned horses presented to the institution due to complaints of active respiratory signs were evaluated by the attending large animal internal medicine clinicians at the time of client-owned horse enrollment. The institution's research herd was evaluated by the same two investigators for all horses (JW and CS). The healthy control group included 11 institution-owned horses, mEA included 4 client-owned and 1 institution-owned horse, and sEA included 13 client-owned and 1 institution-owned horse. Procedures were approved by the University of Tennessee Institutional Animal Care and Use Committee (protocol 2533). Horses from the clinical caseload were enrolled on a volunteer basis with written, informed owner consent, and completed standardized questionnaire. The institution's research herd guidelines for care and use are in accordance with USDA guidelines. All horses had a physical examination (including rebreathing exam), modified 22-point respiratory clinical score (Supplementary Table 1) (41), CBC and fibrinogen, and bronchoalveolar lavage at the time of enrollment.

Horses were retrospectively classified based upon history, clinical presentation, blood work, and BAL cytology. Table 1 contains the diagnostic criteria for each experimental group, in part defined using consensus guidelines stating that airway disease is present, regardless of the procedure, when BALF cytology values are >10% neutrophils, >5% mast cells and >5% eosinophils (1, 42–46). Horses were excluded if < 1 year of age, history of systemic or respiratory disease (besides suspected asthma) in the past 3 months, fever, or corticosteroid (systemic or intranasal) administration initiated within or having significant dosage changes within 2 weeks of enrollment. Healthy horses were defined by a history of no respiratory disease in the last 3 months, normal physical exam, a complete blood count and plasma fibrinogen concentration not consistent with inflammation, and with a normal BALF cytology (< 10% neutrophils, ≤ 1% eosinophils, ≤ 2% mast cells, and ≤ 50% lymphocytes). Asthmatic horses were defined by a history and physical exam findings consistent with asthma, and abnormal BALF cytology. Clinical signs supportive of asthma included cough, increased respiratory rate and effort, poor performance/exercise intolerance, and serous, mucoid, or mucopurulent nasal discharge at rest and/or at exercise. Thoracic auscultation of horses with asthma at rest or during rebreathing exam may reveal wheezes, crackles, cough, tracheal rattle, and/or a prolonged recovery from a rebreathing exam. A complete blood count and plasma fibrinogen concentration was performed at time of enrollment on suspected asthmatic horses to screen for systemic inflammation. A fibrinogen concentration >450 mg/dl was considered abnormal. A BALF cytology consistent with mEA or sEA was required at the time of enrollment. Mild/moderate asthma was defined by a BALF cytology ≥10% neutrophils, ≥5% mast cells, and/or ≥5% eosinophil, and sEA BALF cytology ≥25% neutrophils. At the time of patient enrollment, asthmatic horses were allowed in the study if corticosteroids was initiated > 2 weeks prior to enrollment and no significant changes to current dosages were made within 2 weeks of enrollment as the horses were displaying clinical disease at the time of enrollment despite therapy.

**Table 1:** Defined experimental groups.

**Experimental group**	**History and physical exam**	**CBC/fibrinogen**	**BALF cytology**
Healthy	No history of respiratory abnormalities. Normal PE	Normal	< 10% neutrophils, ≤ 1% eosinophils, ≤ 2% mast cells, and ≤ 50% lymphocytes
Mild to moderate asthma	History and/or presenting complaint of occasional cough and/or poor performance	No signs of infection	≥10% neutrophils, ≥5% mast cells, and/or ≥5% eosinophils
	No history or presence of increased respiratory effort on resting PE		
	± abnormalities on rebreathing exam		
Severe asthma	History and/or presenting complaint of episodes of regular to frequent cough and concurrent increased respiratory effort/rate at rest (not explained by infectious cause)	No signs of infection	≥25% neutrophils
	Presence of one or more of the following on PE: increased respiratory rate, increased respiratory effort, abnormal rebreathing exam		

### 2.2. Sample collection

Blood was collected from the jugular vein into K_2_EDTA tubes (Becton, Dickson, and Co., Franklin Lakes, NJ) prior to sedation, and was submitted to the institution's clinical pathology laboratory for CBC determination using an Advia 2120i hematology instrument (Siemens Healthcare Diagnostics, Inc., Tarrytown, NY) and heat-precipitated fibrinogen measurement using an analog Goldberg TS meter clinical refractometer (Reichert Technologies, Buffalo, NY) by a licensed medical technologist.

Bronchoalveolar lavage procedure was performed on standing, sedated horses using a blind technique with a BAL catheter (Mila International, Florence, KY). Sedation for each horse was determined by the attending clinician and consisted of xylazine (0.2–0.5 mg/kg) or detomidine (5–10 μg/kg), combined with butorphanol (10–20 μg/kg) IV. The BAL catheter was passed nasotracheally until wedged. Once wedged, the cuff was inflated and 200 mL of sterile saline was infused and re-aspirated by 60 ml syringe. The first 10 ml of aspirated sample was discarded as dead space, with subsequent BALF collected and pooled for analysis.

### 2.3. BALF cell count and cytologic analysis

BALF clinicopathologic analysis consisted of a total nucleated cell count (TNCC) and cytologic examination of cytocentrifuged BALF. To optimize preservation of cell morphology, BALF was placed in K_2_EDTA tubes for TNCC and cytologic evaluation.

TNCC was performed using a scil Vet ABC hematology analyzer (scil animal care, a divison of Henry Schein Animal Health, Gurnee, IL) by a trained laboratory assistant or medical technologist according to laboratory standard operating procedures. Daily quality control using three concentrations of commercial control material (Minotrol 16 Whole Blood Hematology Control, Scil AnimalCare, a division of henry Schein Animal Health, Gurnee, IL) was performed. The WBC reported by the instrument was reported as TNCC. To prepare BALF for TNCC, 200 μl of well-mixed BALF was placed into a previously prepared hyaluronidase-containing cryo tubes, in order to break down any mucus that could clog the instrument (47, 48). All TNCC measurements were done in duplicate to mitigate measurement imprecision, and average of the duplicate TNCC was reported.

For cytologic evaluation, 100 μl of well-mixed BALF was placed in a cytocentrifuge (Aerospray 7120 Slide Stainer and Cytocentrifuge, Wescor Incorporated, Logan UT). Cytocentrifuged specimens were air-dried and stained with aqueous Wright's stain. A 300-cell differential cell count, or all cells when < 300 cells on a slide, was performed by a blinded clinical pathologist (BF); respiratory epithelial cells were excluded from these differential counts.

### 2.4. BALF preparation for RNA and cytokine analysis

Pooled BALF was refrigerated at 4°C until further processing, within 2 h of collection. To prepare specimens for cytokine and RNA analysis, BALF was strained through sterile 4 × 4 gauze, followed by a 70 μm nylon cell strainer to remove mucus, and then centrifuged. Aliquots of supernatant were made and stored at −80°C until further analysis. Cell pellets were washed (1 × PBS) two additional times and were stored dry at −80°C until further processing.

### 2.5. RNA extraction and gene expression

RNA was extracted from BALF cell pellets using RNeasy Plus Mini Kit (Qiagen, Hilden, Germany) per manufacturer instructions. Quantification and purity of RNA extracted was assessed *via* NanoDrop 2000c Spectrophotometer (Thermo Fisher Scientific, Waltham, MA). RNA integrity was analyzed on a subset of samples using 2100 Bioanalyzer and the RNA 6000 Nano kit (Agilent, Santa Clara, CA). Reverse transcription and cDNA synthesis was performed on 200 ng of RNA using Maxima First Strand cDNA synthesis kit (Thermo Fisher Scientific) and stored at −80°C. Taqman Gene Expression Assays were used for all target genes; β-actin (*actb*), CD117 (*ckit*), tryptase (*tpsb2*), chymase (*cma1*), and carboxypeptidase A3 (*cpa3*) (Applied Biosystems, Foster City, CA) (Supplementary Table 2 for primer information). Real time PCR was performed on 10 ng cDNA, with Taqman Fast Advanced Master Mix (Applied Biosystems). QuantStudio 6 Flex Real-Time PCR system (Applied Biosystems) was used for thermal cycling at recommended settings. Results were normalized to β-actin and relative quantification was performed using 2^−ΔΔCt^ method (33, 34). Plate results were used when β-actin standard deviations were ≤ 0.7. Average standard deviation of all plates run was 0.55.

### 2.6. Multiplex bead immunoassay analysis

BALF supernatant was thawed at room temperature and centrifuged to remove any precipitates formed during thawing. Cytokines/chemokines included in the assay were: interleukin (IL)-2, IL-4, IL-5, IL-17A, CXC motif chemokine ligand (CXCL)-8, interferon gamma (IFN-γ), and tumor necrosis factor alpha (TNF-α). Cytokine concentrations were quantified using an equine-specific Milliplex^®^ Map Magnetic Bead Panel (EMD Millipore, St Louis, MO, USA) according to the manufacturer's instructions using a Luminex^®^ 200 instrument and Luminex xPONENT^®^ software run in duplicate (Luminex, Austin, TX, USA). Dilutional linearity and percent recovered were determined using low cytokine containing BALF spiked with kit cytokine standard.

Data analysis was done using Milliplex Analyst v5.1 software (EMD Millipore). A bead count of at least 50 beads per well was used for inclusion in analysis and individual samples with a coefficient of variation (CV) > 15% were not included. The Analyst^®^ software assigned the lowest detectable concentration for the individual analytes in which the cytokine/chemokine concentrations fell below the lower limit of detection (LLOD) of the assay. Best-fit standard curve for each analyte was used to calculate analyte concentrations. Analyte concentrations for each horse was normalized to BALF volume recovered.

### 2.7. Statistical analysis

Normality of data was assessed using Shapiro-Wilk test. Descriptive data were expressed as median with interquartile range for both normal and non-normally distributed data for consistency. Descriptive data, protease expression, and cytokine concentrations were analyzed using one-way analysis of variance with Tukey's multiple comparisons in normally distributed data, and by Kruskall-Wallis with Dunn's multiple comparisons in non-normally distributed data. Pairwise correlation of variables including age, sex, cell concentrations, gene expression fold changes, and normalized cytokine concentrations was performed, with additional multidimensional scaling (MDS) analysis in order to visualize on a two-dimensional figure the clustering of samples based on age, sex, cell concentrations, gene expression fold changes, and normalized cytokine concentrations. Bray-Curtis distance function was applied to calculate the dissimilarity matrix of dataset, which ignored missing gene expression values in 4 samples. To evaluate which variable contribute the most to sample clustering, an additional principal components analysis (PCA, which is a specific solution of MDS based on Euclidean distance of complete data) on the samples with no missing values was performed, followed by quantification of the contributions of each variable to the first two principal components. Significance was set at *p* ≤ 0.05.

## 3. Results

### 3.1. Study population

A total of 54 horses were sampled. Twenty-four horses were excluded because they did not meet inclusion criteria (Table 1). Final groups were comprised of 11 healthy and 19 asthmatic horses (5 mild/moderate and 14 severe) (Supplementary Table 3). Median age was not significantly different between groups. The age range of horses enrolled was 8–26 years of age. No young racing horses were included, not on purpose, but was not the typical case load of the institution at the time of sampling. Various breeds were represented in each group.

### 3.2. BALF cell count and cytologic analysis

Results of BALF cell count and cytological analysis are described in Table 2 and Supplementary Table 4. The median total nucleated cell count (TNCC) of each group was not statistically different, although 5 outliers in the sEA group had TNCC > 1,000 cell/μl. As expected, based on standard diagnostic criteria, horses with severe asthma had significantly higher percentages of neutrophils compared to healthy horses (*p* < 0.0001). Horses with sEA had lower percentages of lymphocytes in their BALF when compared to healthy or mEA (*p* = 0.01, *p* = 0.0001, respectively). The composition of leukocytes in healthy horses was predominated by macrophages compared to sEA (*p* < 0.0001). Three horses with mEA had eosinophils detected in their BALF. No eosinophils were detected in BALF from any healthy horses or horses with sEA. Mast cells were detected in two mEA horses and one healthy horse.

**Table 2:** Bronchoalveolar lavage fluid cytology results.

**Group**	**TNCC (cell/μl)**	**%**			
		**N**	**L**	**M**	**E**	**MC**
Ctr	250 [200–300]	4.0^*^ [0.0–9.0]	32^¥^ [26–40.0]	66^*^ [55–73]	0	0
sEA	500 [257–3,775]	74.5^*^ [55.25–89.75]	14.5^*¥^ [1.75–29.0]	10.0^*^ [6.75–18.25]	0	0
mEA	400 [250–725]	12.0 [6.5–19.0]	50^*^ [41.5–58.5]	36 [25.5–44.5]	1.0 [0.0–3.0]	0 [0.0–2.0]

BALF characteristics for the three defined groups: TNCC, Neutrophil%, Lymphocyte%, Macrophage%, Eosinophil%, and Mast cell%. Data are expressed as median [IQR]. Ctr, n = 11; sEA, n = 14; mEA, n = 5. sEA, Severe Asthma; mEA, Mild/Moderate Asthma; Ctr, Healthy; TNCC, Total Nucleated Cell Count (average recorded); N, neutrophil; L, lymphocyte; M, macrophage; E, eosinophil; MC, mast cell. Medians in the same column with the same superscript symbol differ significantly from each other.

### 3.3. Mast cell protease gene expression in BALF cell pellet

**Figure 1:**
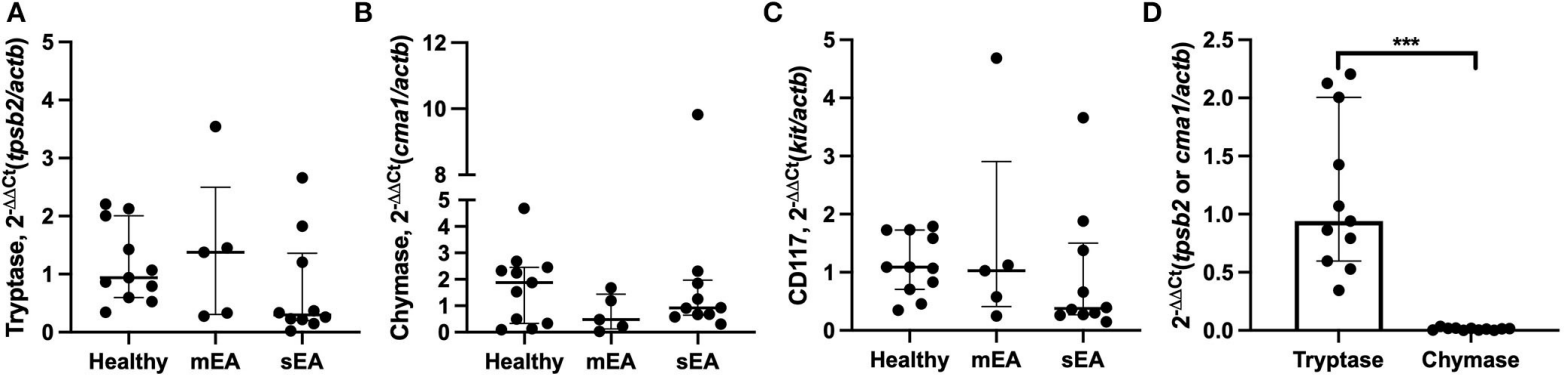
Equine airway mast cells primarily express tryptase with no significant changes in asthmatic horses. Relative gene expression level of **(A)** tryptase (*tpsb2*), **(B)** chymase (*cma1*), and **(C)** CD117 (*ckit*) in BALF cell pellets in healthy (*n* = 11), mild/moderate asthma (*n* = 5), and severe asthma (*n* = 10). All target genes were normalized to the house keeping gene β-actin (*actb*). **(D)** Healthy horse relative expression of detected proteases. Displayed figures represent the median and IQR. sEA, Severe Asthma; mEA, Mild/Moderate Asthma; Ctr, Healthy. *** *p* ≤ 0.001.

To determine whether BALF mast cells have altered protease expression, tryptase, chymase, and/or carboxypeptidase A3 gene expression analysis was performed on BALF cell pellets (Table 3, Figure 1). Four sEA samples were excluded due to poor quality or RNA concentration. First, healthy horse mast cell molecular phenotypes were assessed using relative protease gene expression analysis with normalization to the reference gene, β-actin. Healthy equine airway mast cells express higher amounts of tryptase [fold change: 0.94 (0.60–2.01)] relative to chymase [0.013 (0.002–0.018)] (Figure 1D). Carboxypeptidase A3 was not amplified in any sample, with either primer set identified. Next, evaluation across the three groups was performed. No significant differences in CD117 or protease expression was identified.

**Table 3:** Relative mast cell specific gene expression in BALF cell pellets.

**Group**	**CD117**	**Tryptase**	**Chymase**
Ctr	1.09 [0.70–1.73]	0.94 [0.60–2.01]	1.88 [0.33–2.46]
mEA	1.03 [0.41–2.90]	1.38 [0.30–2.50]	0.48 [0.13–1.44]
sEA	0.38 [0.27–1.50]	0.30 [0.20–1.36]	0.92 [0.65–1.97]

Relative gene expression level of CD117, tryptase, and chymase. Data is represented at the median [IQR] for each group. sEA, Severe Asthma; mEA, Mild/Moderate Asthma; Ctr, Healthy.

### 3.4. Multiplex bead immunoassay analysis (IL-2, IL-4, IL-5, IL-17A, CXCL-8, IFN-γ, TNF-α)

**Figure 2:**
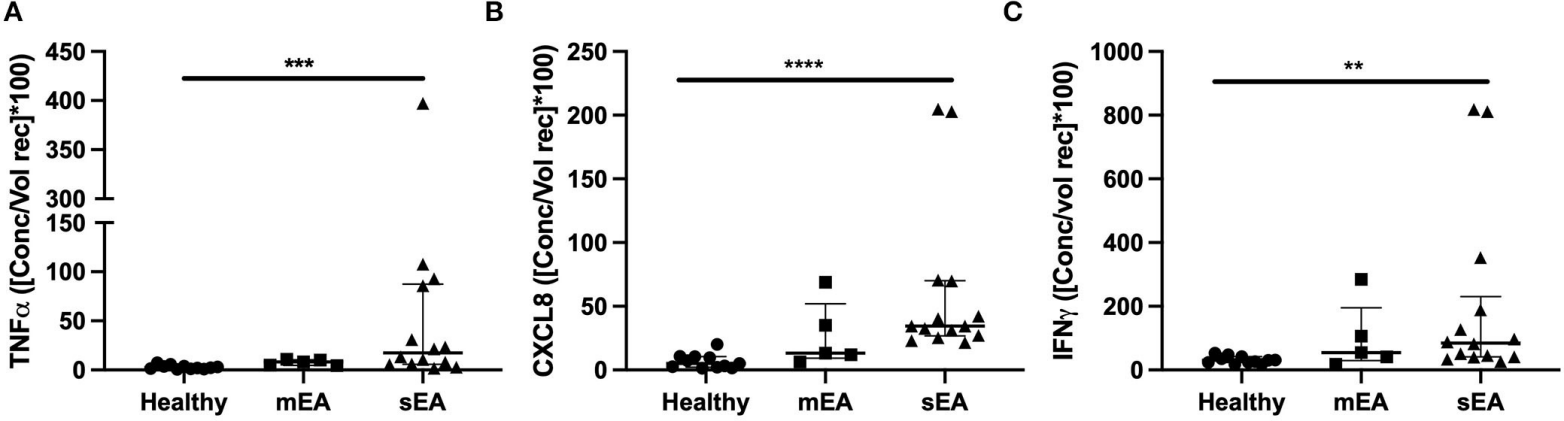
Asthmatic horses have elevated pro-inflammatory cytokines. Normalized cytokine concentrations of **(A)** TNF-α, **(B)** CXCL-8/IL-8, and **(C)** IFN-γ measured *via* multiplex bead immunoassay in healthy (*n* = 11), mild/moderate asthma (*n* = 5), and severe asthma (*n* = 14). Displayed figures represent the median and IQR. sEA, Severe Asthma; mEA, Mild/Moderate Asthma; Ctr, Healthy. **p ≤ 0.01; ***p ≤ 0.001; ****p ≤ 0.0001.

In order to evaluate multiple analytes, maximize limited sample availability, reduce technical errors and optimize efficiency of analysis, multiple bead immunoassay was used for cytokine analysis. Significant differences between groups were found for normalized concentrations for TNF-α, CXCL-8 (formerly known as IL-8), and IFN-γ (Figure 2, Table 4). Analysis showed that horses with severe asthma had significantly higher TNF-α (*p* = 0.0005), CXCL-8 (*p* < 0.0001), and IFN-γ (*p* = 0.006) compared to healthy horses. There were no statistical differences in cytokine concentrations between horses with mEA or sEA, but comparison is limited due to small sample size. Results were not significantly changed by removing horses [4 sEA (horse #6, 9, 11, 12) and 1 mEA (horse #18)] that had a use of corticosteroid therapy with continued clinical signs and potentially corticosteroid-refractory disease (Supplementary Figure 1). Other analytes were extremely low or not detected, therefore normalization and comparison across groups was not performed, but raw values are reported (Supplementary Table 6).

**Table 4:** Normalized cytokine concentrations in BALF supernatant.

**Group**	**IFN-γ normalized**	**CXCL-8 normalized**	**TNF-α normalized**
Ctr	30.11* [23.42–42.16]	5.16* [2.29–10.62]	2.22* [1.14–3.8]
sEA	84.92* [41.07–230.1]	34.44* [26.7–70.12]	17.37* [5.88–87.54]
mEA	54.67 [29.4–195.3]	13.19 [9.21–52.0]	8.23 [4.71–10.51]

Median normalized cytokine concentrations of IFN-γ, CXCL-8, and TNF-α measured *via* multiplex bead immunoassay in healthy (n = 11), mild/moderate asthma (n = 5), and severe asthma (n = 14). sEA, Severe Asthma; mEA, Mild/Moderate Asthma; Ctr, Healthy. Raw concentrations (pg/ml) are normalized to BALF volume recovered (ml) and multiplied by 100. Data are expressed as median [IQR]. Medians in the same column with the same superscript symbol differ significantly from each other.

The equine multiplex bead immunoassay has been used to analyze other more common samples, such as serum, but to the authors' knowledge has not been used to evaluate BALF; therefore, further validation of the assay was performed. A spike and recovery assay using the kit standard and low cytokine concentration BALF was performed, which found the percent recovered was lowest for IL-17A and high at low concentrations for IL-4 and TNF-α (Supplementary Table 7). Additionally, dilutional linearity was achieved for all analytes (Supplementary Figure 2).

### 3.5. Analysis of similarity between samples

**Figure 3:**
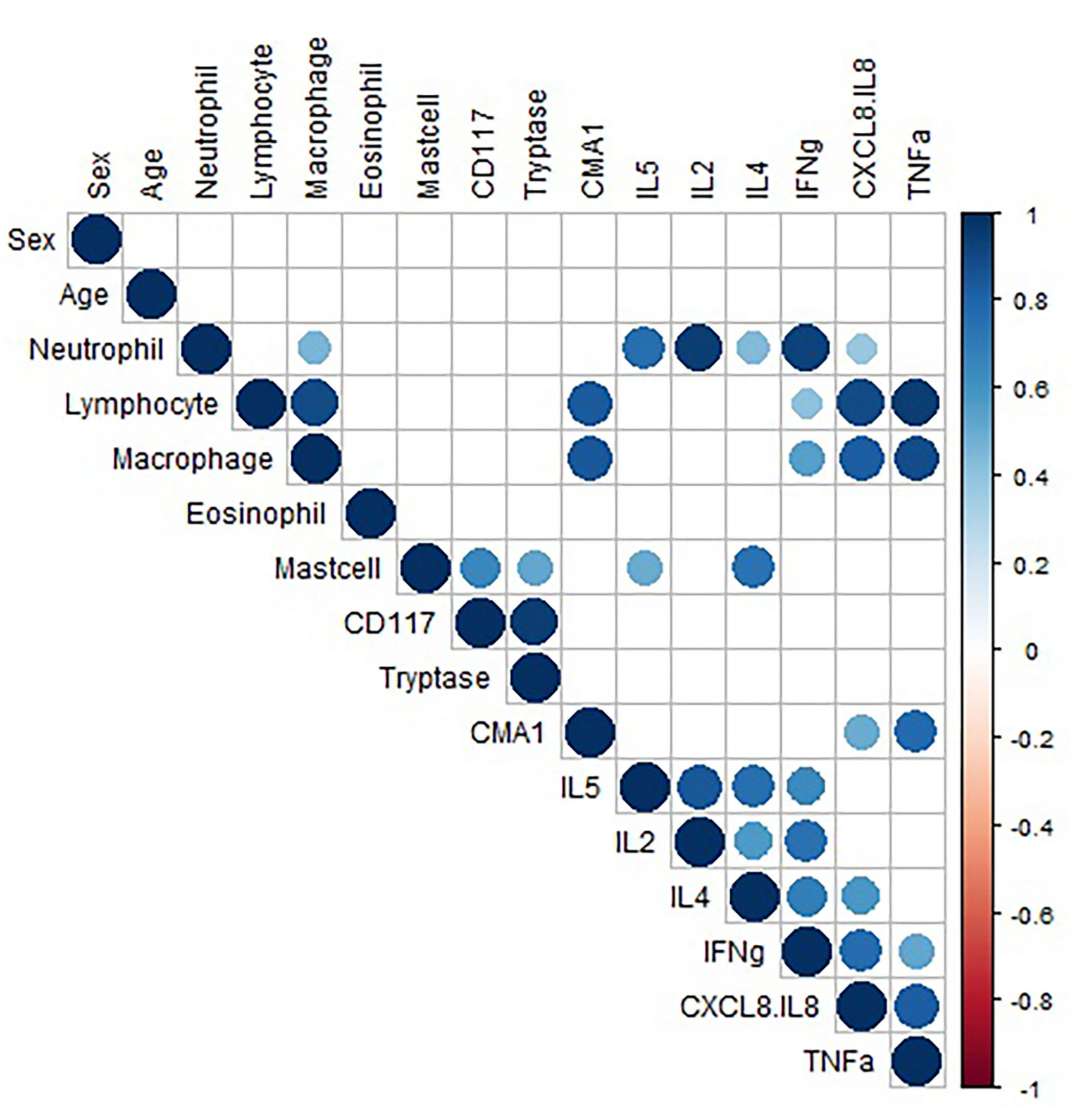
Correlation data. Pairwise correlation of variable. Only significant (*p* < 0.05) correlations are shown. The size of the circle represents the strength of the correlation, and the color represents directionality. The variables are hierarchically clustered based on their correlations.

Pairwise correlation of data was performed (Figure 3). Analysis showed a strong positive correlation between CD117 and tryptase expression (*r* = 0.94), suggesting mast cells primarily express tryptase. Pairwise correlation analysis of the consistently detected cytokines (CXCL-8, IFN-γ, and TNF-α) demonstrated a positive correlation between neutrophil concentration and CXCL-8 and IFN-γ concentrations (Pearson's *r* = 0.38 and 0.93, respectively). Neutrophil concentration showed weak positive correlation with TNF-α (*r* = 0.36), but this association was not statistically significant (*p* = 0.06). CXCL-8 and TNF-α showed a strong positive correlation with lymphocyte and macrophage concentration. The data do not allow interpretation of which cells are making the cytokines, the strong correlations warrant further investigation regarding production and function. While other cytokines also showed significant correlations (IL-5, IL-2, and IL-4), these are hard to interpret due to low to minimal detection. Additionally, significant correlations with mast cell concentration are difficult to interpret due to only 3 horses (2 mEA and 1 healthy) having detectable mast cells.

**Figure 4:**
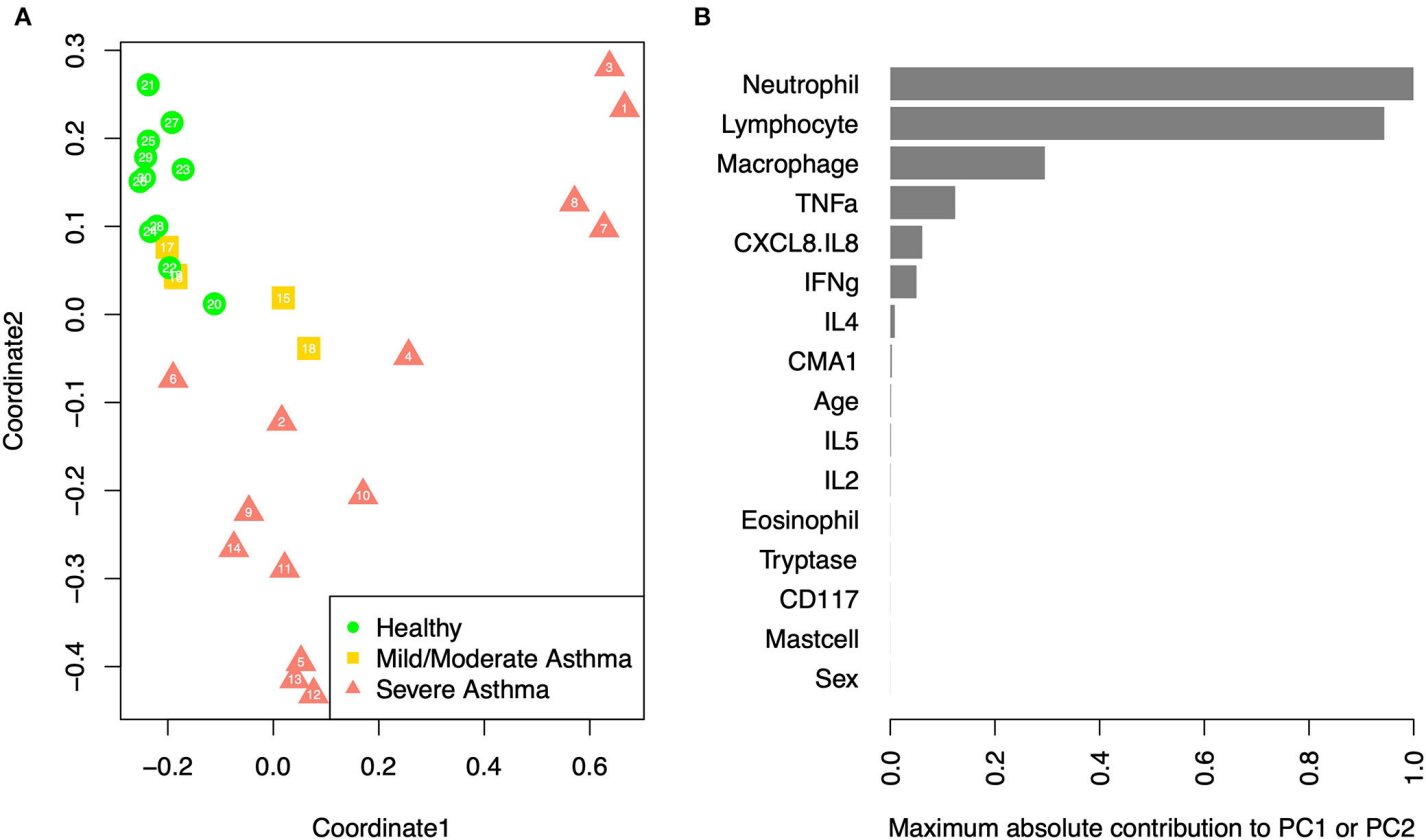
PCA/MDS. **(A)** Multidimensional scaling of the samples based on age, sex, cell percentages, gene expression fold changes, and cytokine concentrations. Each sample is labeled with its anonymous numeric identifier. Asthma status is indicated by gray scale and symbol. **(B)** The maximum contribution to first two principal components from each variable, which shows the relative importance of each variable in driving the observed clustering pattern.

Multidimensional scaling (MDS) analysis showed separation between samples from horses with sEA, mEA, and healthy horses (Figure 4). The healthy samples and mEA samples did not form distinct clusters, but a gradual trend from healthy to mEA to sEA was observed on both axes of the two-dimensional projection. Further principal component analysis on samples with complete data showed that the first two principal components (PCs) explained the majority of the variance, with the first PC explaining 99.97%, and the second PC explaining 94.38% of the variance. The absolute value of the loadings of the first two PCs quantified the contribution of each variable to the observed clustering pattern. By ranking the variables on the maximum absolute loadings from PC1 and PC2, neutrophil concentration and lymphocyte concentration were the main drivers of the observed clusters. While lymphocyte percent decreases in severely asthmatic horses due to pronounced increase in neutrophil percentage, when taking into account cell concentrations, lymphocyte concentration increases alongside neutrophils.

## 4. Discussion

To our knowledge, this study is the first to examine both BALF supernatant cytokine/chemokine concentrations and mast cell protease expression in BALF isolated cells in horses with asthma. These results add to our understanding of the pathophysiology of asthma, while also identifying areas for further investigation.

Further investigation of mast cell phenotypes in horses is supported by human studies in which mast cells and their type is associated with asthma pathology. Increased numbers of MC_T_ in smooth muscle of subjects with asthma have been associated with airway hyper-reactivity (32, 49). Additionally, an increase in MC_TC_ type has been suggested to be protective of lung function in severe, steroid-dependent human asthmatics, which may be associated with corticosteroids ability to decrease MC_T_ numbers (32, 50). Molecular analysis of human lung samples revealed that tryptase and carboxypeptidase A3 were among the most differentially expressed genes in asthmatics vs. control, with Type 2 high asthma associated with expression of tryptase and carboxypeptidase A3, but not chymase (51, 52). Taken together this suggests the importance of continued investigation of mast cell phenotype in relation to asthma type, pathogenesis, and response to therapy in equine asthma.

In healthy horses and those with neutrophilic asthma, this study demonstrates that mast cells in equine BALF express both tryptase and chymase mRNA, but fail to express carboxypeptidase A3. These results suggest the molecular phenotype of healthy equine airway mast cells is primarily tryptase-expressing with low levels of chymase expression and potentially no expression of carboxypeptidase A3. Carboxypeptidase A3 was not amplified in any sample with either primer set utilized. It is currently unclear whether this was due to a lack of carboxypeptidase A3 gene expression in airway mast cells or lack of primary performance. Due to the limited sample size of horses with mastocytic asthma, we were unable to fully evaluate molecular mast cell phenotypes in this group, but it warrants further investigation. Evaluation of mast cell molecular phenotypes was based on previous human studies in which they utilized β-actin as the reference gene; therefore β-actin was used as the reference gene in this study, but recent work in humans and horses has suggested other more suitable reference genes and this will be pursued in further investigations (53, 54). Previous work evaluating mast cell types in horses have investigated lung tissue samples, but not BALF, and reports have been conflicting. A report comparing types in sEA vs. control after moldy hay challenge found no MC_TC_, although severely asthmatic horses had significantly more MC_C_ in the wall of the bronchi (39). In contrast to this report, another study found increased tryptase expression in lung tissue from asthmatic horses vs. control (40). BALF IgE-bearing cells have been evaluated in asthmatic horses and were not different from control horses, although this is not mast cell-specific and did not evaluate mast cell protease expression (55). A very recent study utilized a technique to isolate mast cells from BALF of horses to generate about a 93% pure mast cell population (56). This population of cells then underwent transcriptome analysis and mast cell phenotyping. These results indicated that equine airway mast cells solely express tryptase, with no chymase or carboxypeptidase detected. These results were not further supported by real time PCR. Additionally, the population of cells expressed high levels of MHC class II molecules, further supporting a potential role for mast cells in airway inflammation. The current study suggests mast cell protease dysregulation does not occur in the horses sampled here, although sample size is limited and did not include a group with consistently identified mast cells upon BALF cytological evaluation. It is currently unknown whether inclusion of a mastocytic asthma group would affect these findings or whether a mastocytic asthma subtype within mild to moderate asthma is warranted.

Utilizing a multiplex bead immunoassay allowed simultaneous detection of a panel of cytokines/chemokine concentrations in BALF supernatant. In this study, cytokine/chemokine concentrations in BALF revealed elevated TNF-α and CXCL-8 in sEA horses; therefore, these are potential targets for further subclassification and treatment in asthma. Elevated TNF-α and CXCL-8 parallels findings in human asthma in which asthmatics had increased TNF-α and CXCL-8 vs. control, and neutrophilic asthma was associated with further increased CXCL-8 (57, 58). Our correlation data did not identify a significant correlation with age and protein concentrations, although the concept of inflamm-aging is likely pertinent, especially when evaluating a larger sample size and age ranges. Use of corticosteroids can decrease inflammatory cytokine production, although human asthmatics can be corticosteroid-refractory, which is also likely true in equine asthma. When removing the horses who appeared corticosteroid-refractory, results were not significantly different; therefore, they were left in the overall analysis. In future large-scale studies to further support these initial findings we hope to include “corticosteroid-refractory” EA patients as a separate group. A caveat to the immunoassay used in this study is that a few analytes were poorly detected; therefore, assays having greater analytical sensitivity would be beneficial to define the type of immune response more clearly at the protein level.

We utilized multidimensional scaling to integrate clinical data, cytokine/chemokine concentrations, and mast cell protease expression to investigate potential biomarkers of equine asthma. This analysis indicated that neutrophil and lymphocyte concentrations are key drivers of equine asthma differences, particularly in severe equine asthma. The BAL cytology of sEA is characteristically neutrophilic, with percentages >25%. These results further support the actual concentration increasing, making both neutrophils and lymphocytes potential targets for treatment. Corticosteroids can reduce inflammation by general mechanisms such as inhibiting gene expression of inflammatory cytokines, but targeted cellular therapy against neutrophil/lymphocyte ratios may be more effective, such as reducing CXCL-8, which is key to recruiting and activating neutrophils.

The primary limitation of this study is small sample size, especially that of the mEA in which elevations in mast cell numbers are appreciated. Future directions are aimed at enrolling horses with mastocytic mEA. The institution's case load was skewed toward older horses with severe asthma. Including a younger population of athletic horses with mEA and specifically mastocytic mEA is needed. Another limitation is the evolving definitions of equine asthma. It is very likely that both mEA and sEA have several endotypes and phenotypes. Historically sEA has been typified as those horses with a “heave line” at rest. While sEA horses can have profound clinical signs at rest, they can also experience remission in which their disease becomes subclinical while their neutrophilic inflammation remains (42). The neutrophil driven sEA is reflected in BALF cytology, with neutrophil percent's >25%. In human asthma they use the term severe asthma solely for those that do not respond to therapy despite adherence to optimized therapeutics, but this is not how the veterinary community uses the term currently (59). Evaluating each horse's history, clinical signs, physical examination and diagnostic testing included in this study we can see that respiratory signs can vary between sEA and mEA but the grading of clinical signs is higher in sEA. All cases at the time of evaluation were displaying active clinical signs, although some of the mild to moderate cases are subtle and only displayed signs during exercise. A scoring system or disease severity score that can work across EA types is needed, as well as a consensus on BALF cytological reference ranges for healthy, mEA and sEA. “Gray zones” are present for all the major inflammatory cell types, for example 6–9% neutrophils, 2–4% eosinophils, and 3–4% mast cells. Clinically the neutrophil percent in BALF is the factor that typically delineates between normal and abnormal, as eosinophils and mast cells are innately in lower numbers in normal horses. Lung function testing would give additional objective data for diagnosis, but this is not widely available in non-research setting for client-owned horses. Adult horses were included in this study and while the mean age was not statistically different across groups, this was a small sample size. Aged, geriatric healthy horses have been noted to have changes in BALF cytological evaluations, including increased in lymphocytes and a decreased in macrophages, potentially influencing BALF cytological interpretations (60). The concept of inflamm-aging in horses is also pertinent when interpreting results regarding inflammatory cytokines/chemokines concentrations as elevations in inflammatory proteins has been noted in systemic samples, but how exactly it is involved in airway inflammation is not clear (61, 62).

Innate variations of a blind BALF collection, such as the location sampled or volume of return, even when performed in the same manner, could have influenced results. Evaluation of protein concentrations in BALF supernatant does not have a universally accepted method of normalizing the cytokine/chemokine concentrations across patients. Further, although normalization of analyte concentration to fluid volume recovered during the BAL procedure has been recommended, it is not consistently employed in the literature (63). Our results were normalized to BALF volume recovered. Additional manipulation of the supernatant, such as concentrating techniques, may result in protein loss and inconsistent concentrating due to technical error; therefore, if possible, not manipulating the samples is ideal (64, 65). The analysis of cytokine/chemokine levels in asthma can be done at the gene expression level or protein concentration. In this study we elected to analyze protein concentrations, because changes at the mRNA level are not always reflected at the protein level, for example, due to post-transcriptional modifications.

Ideally mast cell protease expression would have been performed on a homogenous cell population. Tryptase and chymase are primarily made by mast cells, although mast cell protease knockout models have suggested they may also be produced by basophils. However, to our knowledge, basophils have not been identified in equine BALF and were not identified in our samples (66–69). As further evidence, in our study, CD117 expression was highly correlated with tryptase expression levels, therefore the tryptase gene expression is likely to be of mast cell origin, since basophils do not retain CD117 expression once differentiated (70, 71). CD117 can be expressed on differentiated type 2 innate lymphoid cells, however these cells have not yet been described in the horse (72). While we would have preferred mast cell specific analysis of cell protease expression, mast cell isolation techniques and/or flow cytometric analysis of mast cells requires further optimization and will be a part of our ongoing investigations.

This study documented differences in cytokine/chemokine profiles and BALF cell types in asthmatic and healthy horses and provides a basis for further study of these findings. We propose that further evaluation of protease and cytokine/chemokine profiles in larger cohorts of horses, optimally from different geographic locations, will identify biomarkers that support defining equine asthma subtypes based on BALF cellular infiltrate, for example including eosinophilic mEA, mastocytic mEA, neutrophilic mEA, and neutrophilic sEA. We hypothesize that these groups likely have differences that will require specific environmental management and/or treatments for optimal management of the patient.

In conclusion, this study demonstrates that, similar to human patients with asthma, horses with sEA have increases in CXCL-8, TNF-α, and IFN-γ compared to clinically healthy horses. These findings further support the potential role of horses serving as a naturally occurring model of human asthma.

## Data availability statement

The original contributions presented in the study are included in the article/Supplementary material, further inquiries can be directed to the corresponding author.

## Ethics statement

The animal study was reviewed and approved by University of Tennessee Institutional Animal Care and Use Committee. Written informed consent was obtained from the owners for the participation of their animals in this study.

## Author contributions

JW and EL conceived the project and created all other manuscript figures. JW, MH, CS, BF, and EL planned the study design. JW collected and processed samples, performed gene expression and cytokine analysis, analyzed data, wrote the first draft of the manuscript, and processed all UT-CVM samples. BF optimized TNCC procedures and provided guidance on clinicopathologic data. MH, CS, and JW collected UT-CVM samples. MH, CS, BF, and EL edited grant proposals for the support of this research. YL and ZW performed advanced statistical analysis and associated manuscript figures. EL supervised JW and edited all drafts of the manuscript. MH, CS, BF, MS, YL, and ZW edited manuscript. All the authors have read and approved the final manuscript.

## References

[1] Couëtil LL Cardwell JM Gerber V Lavoie J-P Léguillette R Richard EA. Inflammatory airway disease of horses—revised consensus statement. J Vet Intern Med. (2016) 30:503–15. 10.1111/jvim.13824 26806374 PMC4913592

[2] Ferrari CR Cooley J Mujahid N Costa LR Wills RW Johnson ME. Horses with pasture asthma have airway remodeling that is characteristic of human asthma. Vet Pathol. (2018) 55:144–58. 10.1177/0300985817741729 29254472

[3] Bullone M Joubert P Gagné A Lavoie J-P Hélie P. Bronchoalveolar lavage fluid neutrophilia is associated with the severity of pulmonary lesions during equine asthma exacerbations. Equine Vet J. (2018) 50:609–15. 10.1111/evj.12806 29341228

[4] Pirie RS. Recurrent airway obstruction: a review. Equine Vet J. (2014) 46:276–88. 10.1111/evj.12204 24164473

[5] Hotchkiss JW Reid SW Christley RM. A survey of horse owners in Great Britain regarding horses in their care. Part 2: Risk factors for recurrent airway obstruction. Equine Vet J. (2007) 39:301–8. 10.2746/042516407X180129 17722720

[6] Kuruvilla ME Lee FE Lee GB. Understanding asthma phenotypes, endotypes, and mechanisms of disease. Clin Rev Allergy Immunol. (2019) 56:219–33. 10.1007/s12016-018-8712-1 30206782 PMC6411459

[7] Couetil L Cardwell JM Leguillette R Mazan M Richard E Bienzle D. Equine asthma: current understanding and future directions. Front Vet Sci. (2020) 7:450. 10.3389/fvets.2020.00450 32903600 PMC7438831

[8] Bond S Léguillette R Richard EA Couetil L Lavoie J-P Martin JG. Equine asthma: Integrative biologic relevance of a recently proposed nomenclature. J Vet Intern Med. (2018) 32:2088–98. 10.1111/jvim.15302 30294851 PMC6271326

[9] Hughes KJ Nicolson L Da Costa N Franklin SH Allen KJ Dunham SP. Evaluation of cytokine mRNA expression in bronchoalveolar lavage cells from horses with inflammatory airway disease. Vet Immunol Immunopathol. (2011) 140:82–9. 10.1016/j.vetimm.2010.11.018 21194756

[10] Lavoie JP Cesarini C Lavoie-Lamoureux A Moran K Lutz S Picandet V. Bronchoalveolar lavage fluid cytology and cytokine messenger ribonucleic Acid expression of racehorses with exercise intolerance and lower airway inflammation. J Vet Intern Med. (2011) 25:322–9. 10.1111/j.1939-1676.2010.0664.x 21281348

[11] Beekman L Tohver T Leguillette R. Comparison of cytokine mRNA expression in the bronchoalveolar lavage fluid of horses with inflammatory airway disease and bronchoalveolar lavage mastocytosis or neutrophilia using REST software analysis. J Vet Intern Med. (2012) 26:153–61. 10.1111/j.1939-1676.2011.00847.x 22168153

[12] Richard EA Depecker M Defontis M Leleu C Fortier G Pitel P-H. Cytokine concentrations in bronchoalveolar lavage fluid from horses with neutrophilic inflammatory airway disease. J Vet Intern Med. (2014) 28:1838–44. 10.1111/jvim.12464 25269933 PMC4895612

[13] Montgomery JB Husulak ML Kosolofski H Dos Santos S Burgess H Meachem MD. Tumor necrosis factor-alpha protein concentrations in bronchoalveolar lavage fluid from healthy horses and horses with severe equine asthma. Vet Immunol Immunopathol. (2018) 202:70–3. 10.1016/j.vetimm.2018.06.014 30078601

[14] Giguère S Viel L Lee E MacKay RJ Hernandez J Franchini M. Cytokine induction in pulmonary airways of horses with heaves and effect of therapy with inhaled fluticasone propionate. Vet Immunol Immunopathol. (2002) 85:147–58. 10.1016/S0165-2427(01)00420-2 11943316

[15] Lavoie JP Maghni K Desnoyers M Taha R Martin JG Hamid QA. Neutrophilic airway inflammation in horses with heaves is characterized by a Th2-type cytokine profile. Am J Respir Crit Care Med. (2001) 164:1410–3. 10.1164/ajrccm.164.8.2012091 11704587

[16] Debrue M Hamilton E Joubert P Lajoie-Kadoch S Lavoie J-P. Chronic exacerbation of equine heaves is associated with an increased expression of interleukin-17 mRNA in bronchoalveolar lavage cells. Vet Immunol Immunopathol. (2005) 105:25–31. 10.1016/j.vetimm.2004.12.013 15797472

[17] Padoan E Ferraresso S Pegolo S Castagnaro M Barnini C Bargelloni L. Real time RT-PCR analysis of inflammatory mediator expression in recurrent airway obstruction-affected horses. Vet Immunol Immunopathol. (2013) 156:190–9. 10.1016/j.vetimm.2013.09.020 24176614

[18] Tessier L Côté O Clark ME Viel L Diaz-Méndez A Anders S. Impaired response of the bronchial epithelium to inflammation characterizes severe equine asthma. BMC Genomics. (2017) 18:708. 10.1186/s12864-017-4107-6 28886691 PMC5591550

[19] Bond SL Hundt J Leguillette R. Effect of injected dexamethasone on relative cytokine mRNA expression in bronchoalveolar lavage fluid in horses with mild asthma. BMC Vet Res. (2019) 15:397. 10.1186/s12917-019-2144-x 31694631 PMC6833259

[20] Hansen S Otten ND Birch K Skovgaard K Hopster-Iversen C Fjeldborg J. Bronchoalveolar lavage fluid cytokine, cytology and IgE allergen in horses with equine asthma. Vet Immunol Immunopathol. (2019) 220:109976. 10.1016/j.vetimm.2019.109976 31786444

[21] Hue E Orard M Toquet M-P Depecker M Couroucé A Pronost S. Asymmetrical pulmonary cytokine profiles are linked to bronchoalveolar lavage fluid cytology of horses with mild airway neutrophilia. Front Vet Sci. (2020) 7:226. 10.3389/fvets.2020.00226 32391392 PMC7193537

[22] Ainsworth DM Wagner B Franchini M Grünig G Erb HN Tan J-Y. Time-dependent alterations in gene expression of interleukin-8 in the bronchial epithelium of horses with recurrent airway obstruction. Am J Vet Res. (2006) 67:669–77. 10.2460/ajvr.67.4.669 16579761

[23] Sheats MK Davis KU Poole JA. Comparative review of asthma in farmers and horses. Curr Allergy Asthma Rep. (2019) 19:50. 10.1007/s11882-019-0882-2 31599358 PMC9116535

[24] Bullone M Lavoie JP. Asthma “of horses and men”—how can equine heaves help us better understand human asthma immunopathology and its functional consequences? Mol Immunol. (2015) 66:97–105. 10.1016/j.molimm.2014.12.005 25547716

[25] Leclere M Lavoie-Lamoureux A Lavoie JP. Heaves, an asthma-like disease of horses. Respirology. (2011) 16:1027–46. 10.1111/j.1440-1843.2011.02033.x 21824219

[26] Wenzel SE Schwartz LB Langmack EL Halliday JL Trudeau JB Gibbs RL. Evidence that severe asthma can be divided pathologically into two inflammatory subtypes with distinct physiologic and clinical characteristics. Am J Respir Crit Care Med. (1999) 160:1001–8. 10.1164/ajrccm.160.3.9812110 10471631

[27] Wu W Bang S Bleecker ER Castro M Denlinger L Erzurum SC. Multiview cluster analysis identifies variable corticosteroid response phenotypes in severe asthma. Am J Respir Crit Care Med. (2019) 199:1358–67. 10.1164/rccm.201808-1543OC 30682261 PMC6543720

[28] Lambrecht BN Hammad H Fahy JV. The cytokines of asthma. Immunity. (2019) 50:975–91. 10.1016/j.immuni.2019.03.018 30995510

[29] Salvermoser M Zeber K Boeck A Klucker E Schaub B. Childhood asthma: novel endotyping by cytokines, validated through sensitization profiles and clinical characteristics. Clin Exp Allergy. (2021) 51:654–65. 10.1111/cea.13858 33650157

[30] Gordon ED Simpson LJ Rios CL Ringel L Lachowicz-Scroggins ME Peters MC. Alternative splicing of interleukin-33 and type 2 inflammation in asthma. Proc Natl Acad Sci USA. (2016) 113:8765–70. 10.1073/pnas.1601914113 27432971 PMC4978244

[31] Fahy JV. Type 2 inflammation in asthma—present in most, absent in many. Nat Rev Immunol. (2015) 15:57–65. 10.1038/nri3786 25534623 PMC4390063

[32] Balzar S Fajt ML Comhair SAA Erzurum SC Bleecker E Busse WW. Mast cell phenotype, location, and activation in severe asthma. Data from the Severe Asthma Research Program. Am J Respir Crit Care Med. (2011) 183:299–309. 10.1164/rccm.201002-0295OC 20813890 PMC3056228

[33] Wang G Baines KJ Fu JJ Wood LG Simpson JL McDonald VM. Sputum mast cell subtypes relate to eosinophilia and corticosteroid response in asthma. Eur Respir J. (2016) 47:1123–33. 10.1183/13993003.01098-2015 26699720

[34] Berthon BS Gibson PG Wood LG MacDonald-Wicks LK Baines KJ. A sputum gene expression signature predicts oral corticosteroid response in asthma. Eur Respir J. (2017) 49:1700180. 10.1183/13993003.00180-2017 28663317

[35] Fricker M Qin L Niessen N Baines KJ McDonald VM Scott HA. Relationship of sputum mast cells with clinical and inflammatory characteristics of asthma. Clin Exp Allergy. (2020) 2020:696–707. 10.1111/cea.13609 32291815

[36] Irani AA Schechter NM Craig SS DeBlois G Schwartz LB. Two types of human mast cells that have distinct neutral protease compositions. Proc Natl Acad Sci USA. (1986) 83:4464–8. 10.1073/pnas.83.12.4464 3520574 PMC323754

[37] Irani AM Goldstein SM Wintroub BU Bradford T Schwartz LB. Human mast cell carboxypeptidase. Selective localization to MCTC cells. J Immunol. (1991) 147:247–53. 2051021

[38] Beil WJ Pammer J. In situ detection of the mast cell proteases chymase and tryptase in human lung tissue using light and electron microscopy. Histochem Cell Biol. (2001) 116:483–93. 10.1007/s00418-001-0339-1 11810190

[39] van der Haegen A Künzle F Gerber V Welle M Robinson NE Marti E. Mast cells and IgE-bearing cells in lungs of RAO-affected horses. Vet Immunol Immunopathol. (2005) 108:325–34. 10.1016/j.vetimm.2005.06.005 16040130

[40] Dacre KJ McGorum BC Marlin DJ Bartner LR Brown JK Shaw DJ. Organic dust exposure increases mast cell tryptase in bronchoalveolar lavage fluid and airway epithelium of heaves horses. Clin Exp Allergy. (2007) 37:1809–18. 10.1111/j.1365-2222.2007.02857.x 17956586

[41] Lavoie J-P Bullone M Rodrigues N Germim P Albrecht B von Salis-Soglio M. Effect of different doses of inhaled ciclesonide on lung function, clinical signs related to airflow limitation and serum cortisol levels in horses with experimentally induced mild to severe airway obstruction. Equine Vet J. (2019) 51:779–86. 10.1111/evj.13093 30854685 PMC7379559

[42] Wasko AJ Barkema HW Nicol J Fernandez N Logie N Léguillette R. Evaluation of a risk-screening questionnaire to detect equine lung inflammation: results of a large field study. Equine Vet J. (2011) 43:145–52. 10.1111/j.2042-3306.2010.00150.x 21592207

[43] Kang H Bienzle D Lee GKC Piché É Viel L Odemuyiwa SO. Flow cytometric analysis of equine bronchoalveolar lavage fluid cells in horses with and without severe equine asthma. Vet Pathol. (2022) 59:91–9. 10.1177/03009858211042588 34521286 PMC8679174

[44] Richard EA Fortier GD Denoix J-M Art T Lekeux PM Van Erck E. Influence of subclinical inflammatory airway disease on equine respiratory function evaluated by impulse oscillometry. Equine Vet J. (2009) 41:384–9. 10.2746/042516409X366121 19562901

[45] Richard EA Fortier GD Lekeux PM Van Erck E. Laboratory findings in respiratory fluids of the poorly-performing horse. Vet J. (2010) 185:115–22. 10.1016/j.tvjl.2009.05.003 19481964

[46] Koblinger K Nicol J McDonald K Wasko A Logie N Weiss M. Endoscopic assessment of airway inflammation in horses. J Vet Intern Med. (2011) 25:1118–26. 10.1111/j.1939-1676.2011.00788.x 21985142

[47] Ekmann A Rigdal ML Gröndahl G. Automated counting of nucleated cells in equine synovial fluid without and with hyaluronidase pretreatment. Vet Clin Pathol. (2010) 39:83–9. 10.1111/j.1939-165X.2009.00203.x 20051063

[48] Berger JT Voynow JA Peters KW Rose MC. Respiratory carcinoma cell lines. MUC genes and glycoconjugates. Am J Respir Cell Mol Biol. (1999) 20:500–10. 10.1165/ajrcmb.20.3.3383 10030849

[49] Brightling CE Bradding P Symon FA Holgate ST Wardlaw AJ Pavord ID. Mast-cell infiltration of airway smooth muscle in asthma. N Engl J Med. (2002) 346:1699–705. 10.1056/NEJMoa012705 12037149

[50] Balzar S Chu HW Strand M Wenzel S. Relationship of small airway chymase-positive mast cells and lung function in severe asthma. Am J Respir Crit Care Med. (2005) 171:431–9. 10.1164/rccm.200407-949OC 15563633

[51] Dougherty RH Sidhu SS Raman K Solon M Solberg OD Caughey GH. Accumulation of intraepithelial mast cells with a unique protease phenotype in T(H)2-high asthma. J Allergy Clin Immunol. (2010) 125:1046–53.e8. 10.1016/j.jaci.2010.03.003 20451039 PMC2918406

[52] Woodruff PG Boushey HA Dolganov GM Barker CS Yang YH Donnelly S. Genome-wide profiling identifies epithelial cell genes associated with asthma and with treatment response to corticosteroids. Proc Natl Acad Sci USA. (2007) 104:15858–63. 10.1073/pnas.0707413104 17898169 PMC2000427

[53] Moermans C Deliege E Pirottin D Poulet C Guiot J Henket M. Suitable reference genes determination for real-time PCR using induced sputum samples. Eur Respir J. (2019) 54:1800644. 10.1183/13993003.00644-2018 31601710

[54] Beekman L Tohver T Dardari R Léguillette R. Evaluation of suitable reference genes for gene expression studies in bronchoalveolar lavage cells from horses with inflammatory airway disease. BMC Mol Biol. (2011) 12:5. 10.1186/1471-2199-12-5 21272375 PMC3039571

[55] Künzle F Gerber V Van Der Haegen A Wampfler B Straub R Marti E. IgE-bearing cells in bronchoalveolar lavage fluid and allergen-specific IgE levels in sera from RAO-affected horses. J Vet Med A Physiol Pathol Clin Med. (2007) 54:40–7. 10.1111/j.1439-0442.2007.00870.x 17359454

[56] Akula S Riihimäki M Waern I Åbrink M Raine A Hellman L. Quantitative transcriptome analysis of purified equine mast cells identifies a dominant mucosal mast cell population with possible inflammatory functions in airways of asthmatic horses. Int J Mol Sci. (2022) 23:13976. 10.3390/ijms232213976 36430453 PMC9692376

[57] Hosoki K Ying S Corrigan C Qi H Kurosky A Jennings K. Analysis of a panel of 48 cytokines in BAL fluids specifically identifies IL-8 levels as the only cytokine that distinguishes controlled asthma from uncontrolled asthma, and correlates inversely with FEV1. PLoS ONE. (2015) 10:e0126035. 10.1371/journal.pone.0126035 26011707 PMC4444276

[58] Yang T Li Y Lyu Z Huang K Corrigan CJ Ying S. Characteristics of proinflammatory cytokines and chemokines in airways of asthmatics: relationships with disease severity and infiltration of inflammatory cells. Chin Med J (Engl). (2017) 130:2033–40. 10.4103/0366-6999.213428 28836545 PMC5586170

[59] Global Initiative for Asthma. Global Strategy for Asthma Management Prevention. GINA (2022). Available online at: www.ginasthma.org

[60] Pacheco AP Paradis MR Hoffman AM Hermida P Sanchez A Nadeau JA. Age effects on blood gas, spirometry, airway reactivity, and bronchoalveolar lavage fluid cytology in clinically healthy horses. J Vet Intern Med. (2014) 28:603–8. 10.1111/jvim.12318 24528225 PMC4857999

[61] Adams AA Breathnach CC Katepalli MP Kohler K Horohov DW. Advanced age in horses affects divisional history of T cells and inflammatory cytokine production. Mech Ageing Dev. (2008) 129:656–64. 10.1016/j.mad.2008.09.004 18926847

[62] Hansen S Baptiste KE Fjeldborg J Horohov DW. A review of the equine age-related changes in the immune system: comparisons between human and equine aging, with focus on lung-specific immune-aging. Ageing Res Rev. (2015) 20:11–23. 10.1016/j.arr.2014.12.002 25497559

[63] Haslam PL Baughman RP. Report of ERS Task Force: guidelines for measurement of acellular components and standardization of BAL. Eur Respir J. (1999) 14:245–8. 10.1034/j.1399-3003.1999.14b01.x 10515395

[64] Lam S LeRiche JC Kijek K. Effect of filtration and concentration on the composition of bronchoalveolar lavage fluid. Chest. (1985) 87:740–2. 10.1378/chest.87.6.740 3996060

[65] Afford SC Stockley RA Kramps JA Dijkman JH Burnett D. Concentration of bronchoalveolar lavage fluid by ultrafiltration: evidence of differential protein loss and functional inactivation of proteinase inhibitors. Anal Biochem. (1985) 151:125–30. 10.1016/0003-2697(85)90061-2 3853960

[66] Heutinck KM ten Berge IJ Hack CE Hamann J Rowshani AT. Serine proteases of the human immune system in health and disease. Mol Immunol. (2010) 47:1943–55. 10.1016/j.molimm.2010.04.020 20537709

[67] Pejler G Abrink M Ringvall M Wernersson S. Mast cell proteases. Adv Immunol. (2007) 95:167–255. 10.1016/S0065-2776(07)95006-3 17869614

[68] Pejler G Rönnberg E Waern I Wernersson S. Mast cell proteases: multifaceted regulators of inflammatory disease. Blood. (2010) 115:4981–90. 10.1182/blood-2010-01-257287 20233968

[69] Reber LL Marichal T Galli SJ. New models for analyzing mast cell functions in vivo. Trends Immunol. (2012) 33:613–25. 10.1016/j.it.2012.09.008 23127755 PMC3572764

[70] Han X Jorgensen JL Brahmandam A Schlette E Huh YO Shi Y. Immunophenotypic study of basophils by multiparameter flow cytometry. Arch Pathol Lab Med. (2008) 132:813–9. 10.5858/2008-132-813-ISOBBM 18466030

[71] Galli SJ Tsai M Wershil BK. The c-kit receptor, stem cell factor, and mast cells. What each is teaching us about the others. Am J Pathol. (1993) 142:9 65–74. 7682764 PMC1886888

[72] Hochdörfer T Winkler C Pardali K Mjösberg J. Expression of c-Kit discriminates between two functionally distinct subsets of human type 2 innate lymphoid cells. Eur J Immunol. (2019) 49:884–93. 10.1002/eji.201848006 30892687

