# Supplementary material for: Initial investigation of molecular phenotypes of airway mast cells and cytokine profiles in equine asthma
Source: Front Vet Sci. 2023 Jan 11;9:997139. doi: 10.3389/fvets.2022.997139 (PMC9875299; doi:10.3389/fvets.2022.997139)
Supplement: Supplementary file 1 [file Data_Sheet_1.docx]

**Supplementary Material**

**1 Supplemental Figures**

**Figure 1. Normalized cytokine concentrations of horses, excluding those on corticosteroids.** Normalized cytokine concentrations of (A) TNF-α, (B) CXCL-8/IL-8, and (C) IFN-ɣ measured via multiplex bead immunoassay in healthy (n=11), mild/moderate asthma (n = 4), and severe asthma (n = 10). One horse in the mEA group, and 4 in the sEA group had been on corticosteroids with continued clinical signs. Excluding these horses did not change overall results. Displayed figures represent the median and IQR. sEA= Severe Asthma, mEA= Mild/Moderate Asthma, Ctr= Healthy. *, p ≤ 0.05 ; **, p ≤ 0.01; ****, p ≤ 0.0001

(A)

(B)

(C)

**Figure 2. Dilutional linearity of individual cytokines.** Dilutional linearity as depicted by expected versus measured with linear regression analysis for all cytokines of interest: (A) IL-2 (B) IL-4, (C) IL-5, (D) IL-17A, (E) TNF-α, (F) IFN-ɣ, (G) CXCL-8. All cytokines show high linear dilution of spiked samples. The kit cytokine standard was used to re-capitulate the standard curve by using the top standard, initially reconstituted in water, to further dilute in BALF supernatant. The points depicted are based on the kit’s standard reference range for each analyte.

(A)

**
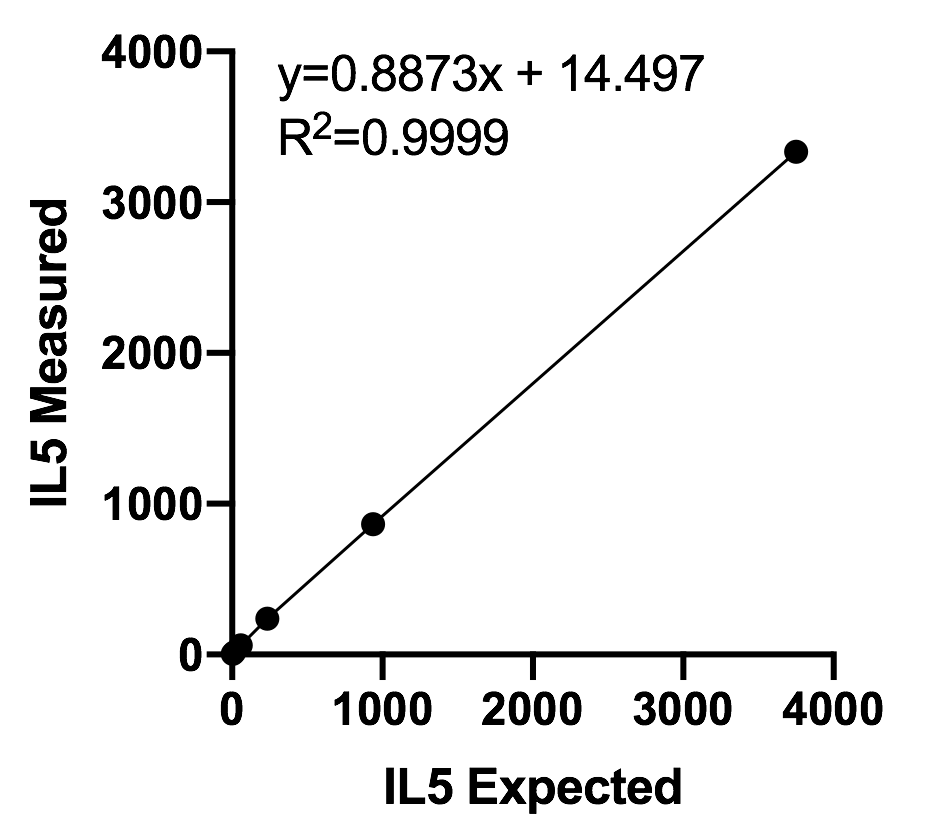
**

(B)

**
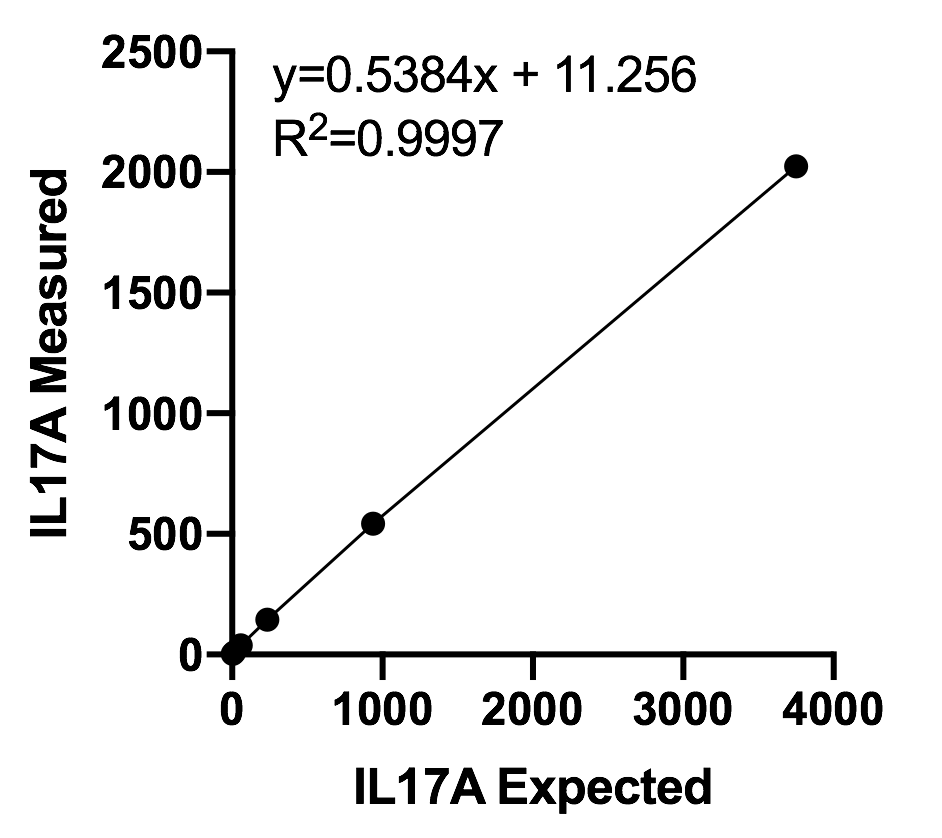
**

(C)

**
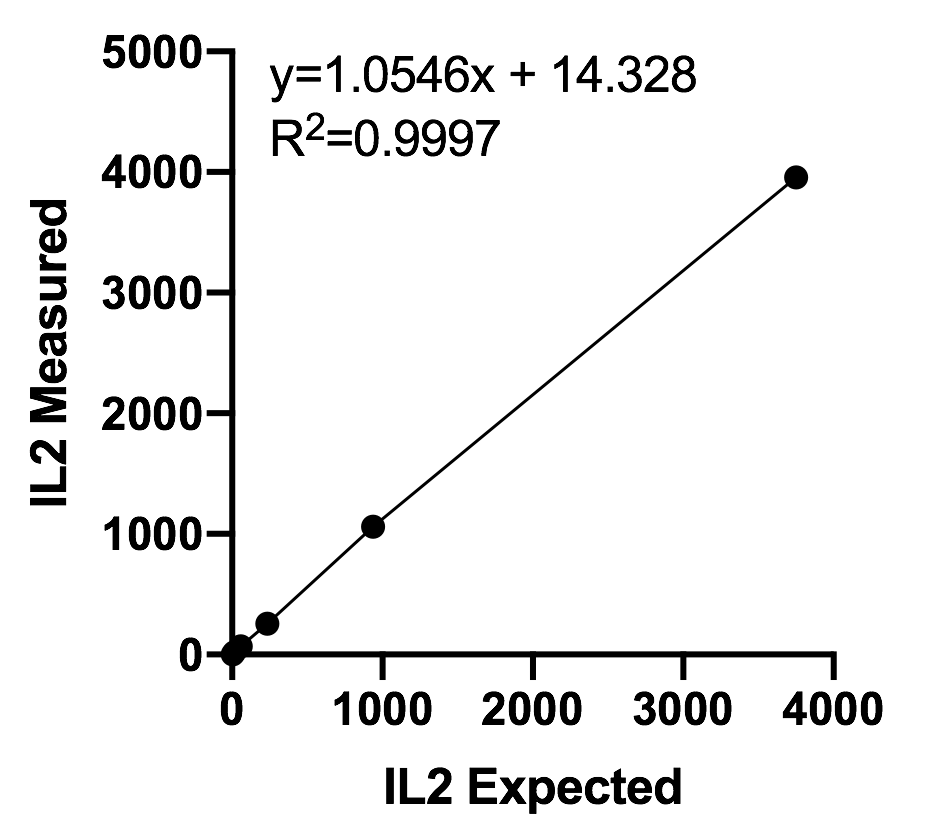
**

(D)

**
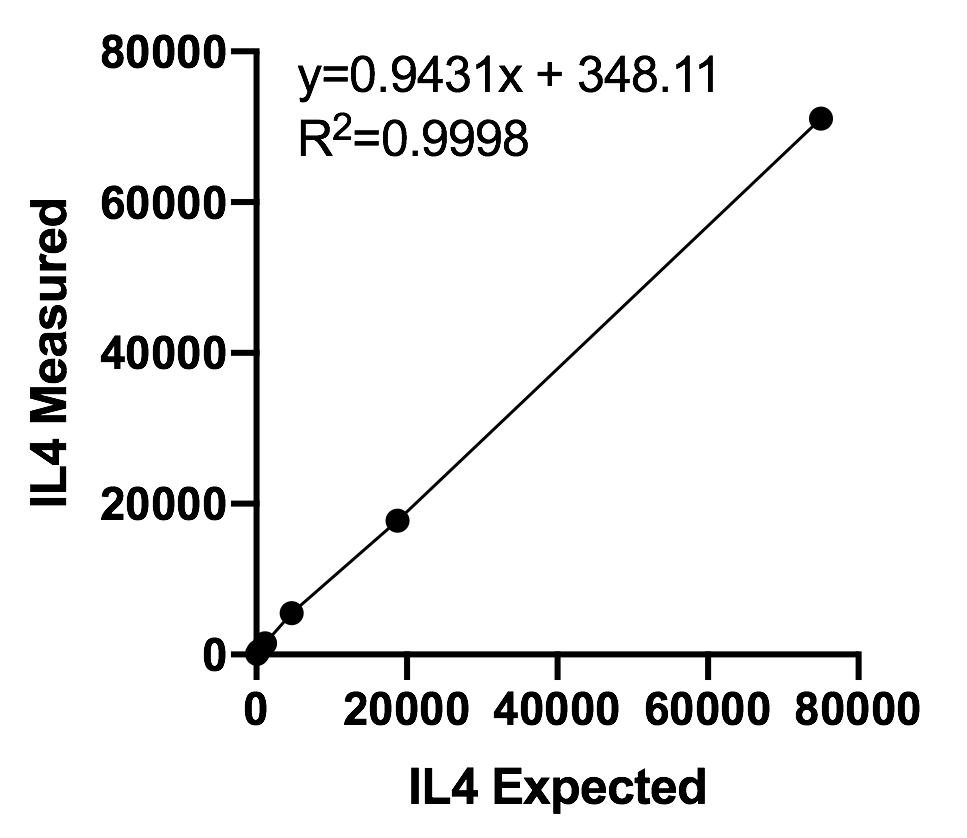
**

(E)

**
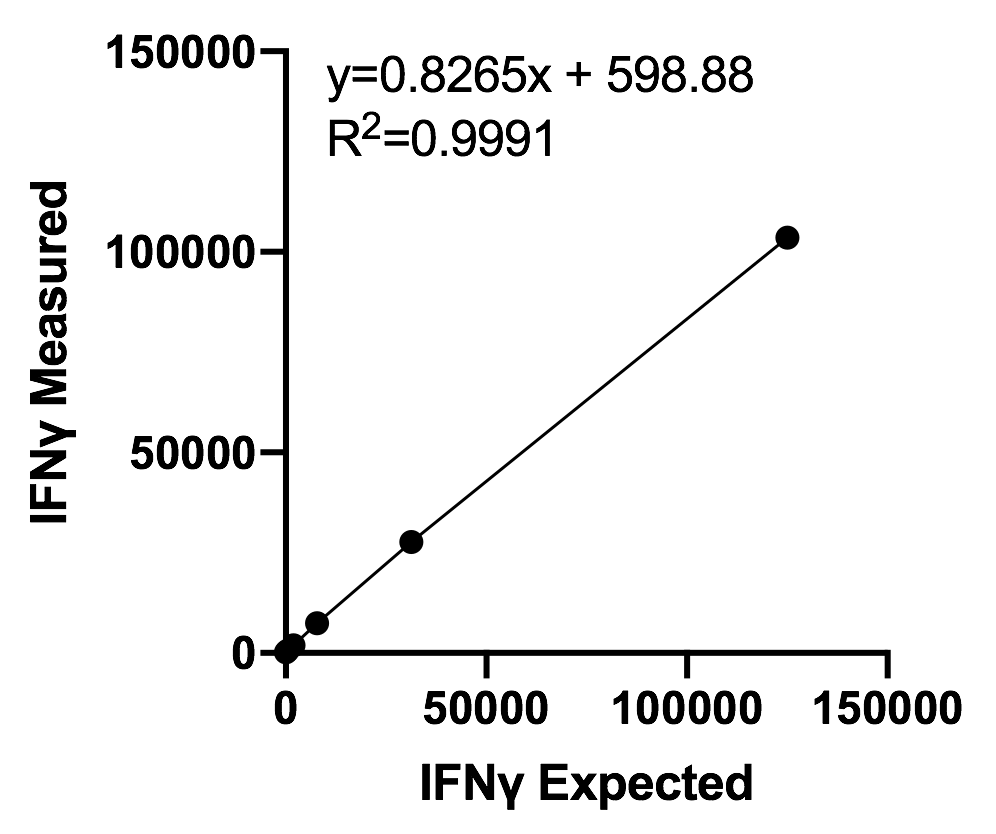
**

(F)

**
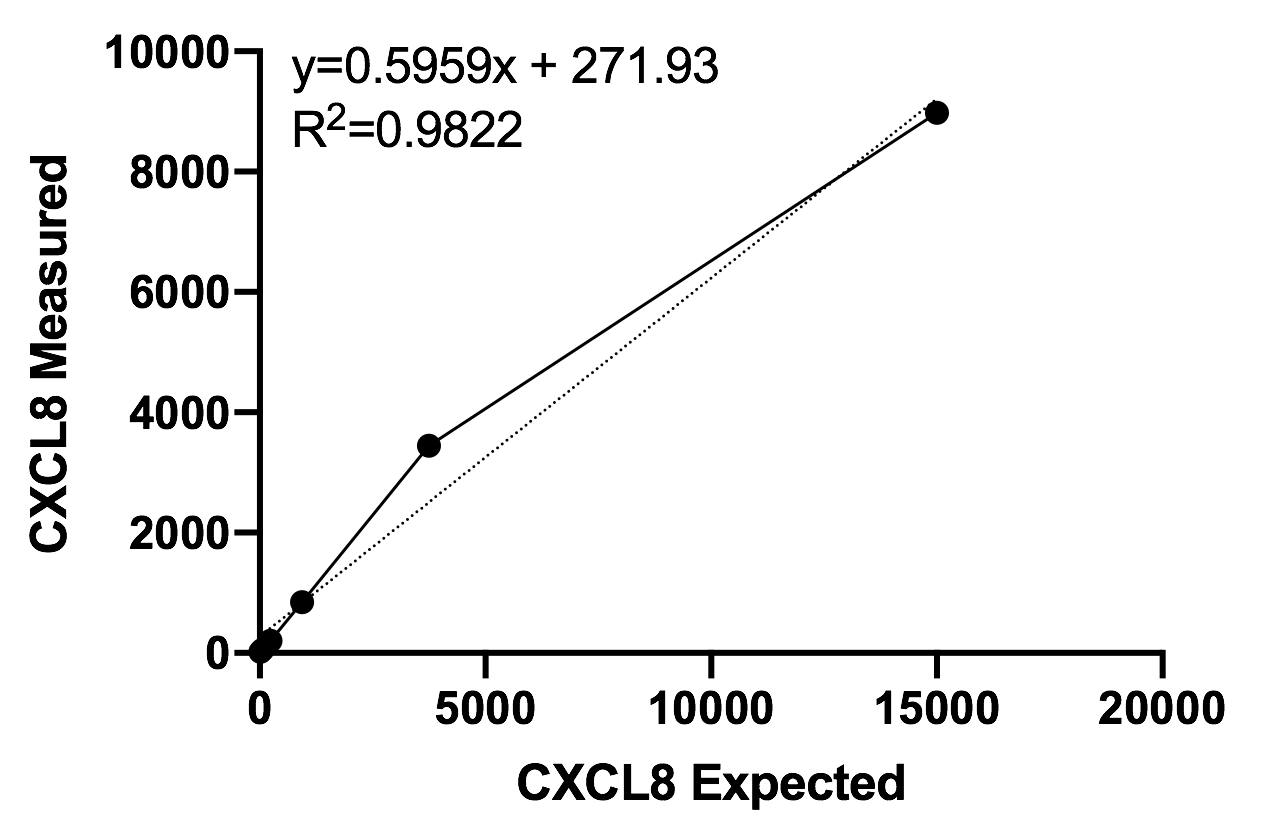
**

(G)

**
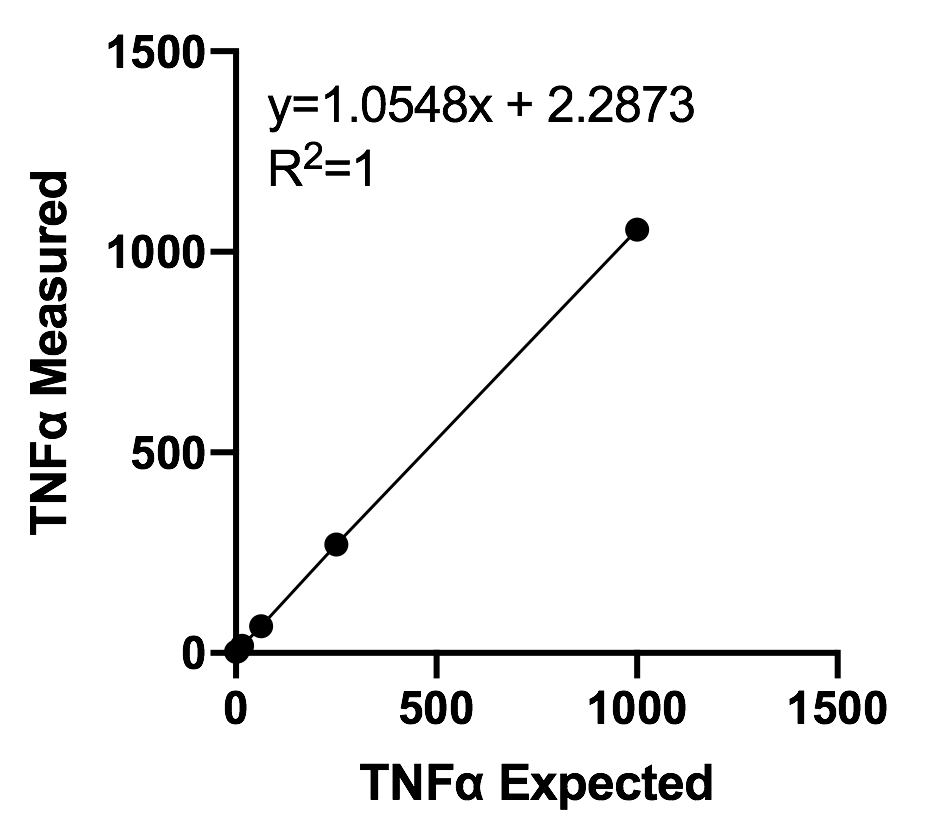
**

**2 Supplemental Tables**

**Supplemental Table 1. Clinical score adapted from Lavoie et al. (41)**

| **Variable** | **Range** | **Description** |
| --- | --- | --- |
| Nasal discharge | 0-3 | 0 none  1 serous  3 mucopurulent |
| Nasal flare (NF) | 0-1 | 0 none  1 present |
| Cough | 0-3 | 0 none  1 inducible by tracheal massage  2 intermittent  3 paroxysmal |
| Respiratory Rate | 0-3 | 0 <16/minute  1 16-20/minute  2 21-30/minute  3 >30/minute |
| Abdominal lift | 0-3 | 0 none  1 mild, perceptible heave line  3 Pronounced, abdominal effort and thorax |
| Tracheal sounds | 0-3 | 0 normal  1 increased in intensity  3 mucous movement |
| Rebreathing exam | 0-2 | 0 normal  1 increased bronchovesicular sounds  2 delayed recovery and/or cough |
| Crackles at rest or during rebreathing exam | 0-2 | 0 normal  2 present |
| Wheezes at rest or during rebreathing exam | 0-2 | 0 normal  2 present |
| **Score sum** | **0-22** | **Sum of all scores above** |

**Supplemental Table 2. Taqman Gene Expression Assay IDs.**

| **Gene Target** | **Assay ID** |
| --- | --- |
| β-actin | Ec04176172_gH |
| CD117 | Ec03469720_m1 |
| Tryptase | Ec03468172_m1 |
| Chymase | ARMFXWU (custom) |
| Carboxypeptidase A3 | APU629N, ARH6ATU (custom) |
| Transforming growth factor-β1 | Ec03468030_m1 |
| Bone morphogenetic protein-7 | Ec04320876_m1 |

**Supplemental Table 3. Individual horse descriptive data.** Experimental groups: history & clinical signs, rebreathing exam, sex, age, & breed for individual horses in each group. *sEA= Severe Asthma, mEA= Mild/Moderate Asthma, Ctr= Healthy; M= Mare, G= Gelding; NSF= No significant findings; MFT= Missouri Fox Trotter, ASH= American Saddle Horse, TWH= Tennessee Walking Horse, H= Holsteiner, QH= Quarter Horse, PF= Paso Fino, TB= Thoroughbred, APH= American Paint Horse, TBx= Thoroughbred cross.

| **Horse** | **Diagnosis** | **Clinical Signs** | **Rebreathing Exam** | **Clinical score** | **Sex** | **Age (yrs)** | **Breed** |
| --- | --- | --- | --- | --- | --- | --- | --- |
| 1 | sEA | Chronic cough | Delayed recovery | 3 | M | 20 | Pony |
| 2 | sEA | Chronic cough, intermittent nasal discharge | NSF | 3 | M | 13 | Not reported |
| 3 | sEA | Chronic cough | Tracheal rattle | 5 | M | 13 | MFT |
| 4 | sEA | Chronic cough, intermittent nasal discharge | Crackles, wheezes | 7 | M | 13 | ASH |
| 5 | sEA | Chronic cough | Crackles, wheezes | 6 | G | 12 | Pony |
| 6 | sEA | Increased respiratory effort | Wheezes | 5 | M | 14 | TWH |
| 7 | sEA | Chronic cough, exercise intolerance | Wheezes | 4 | G | 19 | Not reported |
| 8 | sEA | Chronic cough | NSF | 2 | G | 20 | Arab |
| 9 | sEA | Increased respiratory rate at rest | Wheezes | 6 | G | 14 | TWH |
| 10 | sEA | Chronic cough, intermittent nasal discharge | Crackles, wheezes | 7 | M | 20 | Arab |
| 11 | sEA | Wheezes and increased respiratory effort at rest | Wheezes | 8 | G | 10 | TWH |
| 12 | sEA | Chronic intermittent cough and increased respiratory rate | Wheezes, occasional crackle, increased respiratory effort | 9 | G | 19 | H |
| 13 | sEA | Chronic intermittent cough | Wheezes and worsened cough | 4 | G | 12 | QH |
| 14 | sEA | Cough, increased respiratory effort | Worsened respiratory effort and cough | 5 | G | 17 | QH |
| 15 | mEA | Increased respiratory rate in barn | Increased bronchovesicular sounds | 1 | M | 18 | QH |
| 16 | mEA | Cough, intermittent nasal discharge | Cough upon tracheal palpation, delayed recovery | 5 | G | 13 | QH |
| 17 | mEA | None observed | Delayed recovery | 2 | M | 22 | QH |
| 18 | mEA | Chronic cough | Wheezes | 4 | G | 14 | MFT |
| 19 | mEA | Exercise intolerance, intermittent nasal discharge | Delayed recovery, nasal discharge | 5 | G | 11 | PF |
| 20 | Ctr | None | NSF | 1 | M | 8 | TWH |
| 21 | Ctr | None | NSF | 1 | M | 18 | QH |
| 22 | Ctr | None | NSF | 0 | M | 19 | QH |
| 23 | Ctr | None | NSF | 0 | M | 16 | QH |
| 24 | Ctr | None | NSF | 0 | M | 14 | MFT |
| 25 | Ctr | None | NSF | 0 | M | 17 | QH |
| 26 | Ctr | None | NSF | 1 | M | 26 | APH |
| 27 | Ctr | None | NSF | 1 | M | 16 | TWH |
| 28 | Ctr | None | NSF | 1 | G | 11 | TWH |
| 29 | Ctr | None | NSF | 0 | M | 14 | QH |
| 30 | Ctr | None | NSF | 0 | G | 12 | TB |

**Supplemental Table 4. Individual horse BAL results.** BALF results: fluid volume recovered, % volume recovered, TNCC, neutrophil %, lymphocyte %, macrophage %, eosinophil %, and mast cell % for individual horses in each group. Data is represented at the median [IQR] for each group. *sEA= Severe Asthma, mEA= Mild/Moderate Asthma, Ctr= Healthy; TNCC= Total Nucleated Cell Count (Average recorded); N= neutrophil, L= lymphocyte, M= macrophage, E= eosinophil, MC= mast cell.

|  |  |  |  |  |  | **%** |  |  |
| --- | --- | --- | --- | --- | --- | --- | --- | --- |
| **Horse** | **mLs Fluid Recovered** | **% Volume Recovered** | **TNCC**  **(cell/µL)** | **N** | **L** | **M** | **E** | **MC** |
| **sEA** |  |  |  |  |  |  |  |  |
| 1 | 23 | 11.5 | 13,000 | 70 | 22 | 8 | 0 | 0 |
| 2 | 62 | 31 | 900 | 33 | 40 | 27 | 0 | 0 |
| 3 | 120 | 60 | 49,000 | 98 | 1 | 1 | 0 | 0 |
| 4 | 52 | 26 | 2,000 | 57 | 26 | 17 | 0 | 0 |
| 5 | 24 | 12 | 250 | 81 | 5 | 14 | 0 | 0 |
| 6 | 78 | 39 | 200 | 32 | 32 | 36 | 0 | 0 |
| 7 | 19 | 9.5 | Unable to determine | 97 | 0 | 3 | 0 | 0 |
| 8 | 30 | 15 | 5,550 | 89 | 4 | 7 | 0 | 0 |
| 9 | 58 | 29 | 300 | 50 | 28 | 22 | 0 | 0 |
| 10 | 70 | 35 | 1,150 | 79 | 14 | 7 | 0 | 0 |
| 11 | 40 | 40 | 500 | 69 | 15 | 16 | 0 | 0 |
| 12 | 60 | 60 | 200 | 92 | 2 | 6 | 0 | 0 |
| 13 | 46 | 46 | 300 | 87 | 1 | 12 | 0 | 0 |
| 14 | 81 | 81 | 350 | 59 | 33 | 8 | 0 | 0 |
| ***Median [IQR]*** | 55  [28.5 – 72] | 27.5  [14.25 – 36.0] | 500  [257 – 3775] | 83.0  [57.0 – 94.0] | 10  [1 – 26.5] | 6.5  [4.5 – 16.5] | 0 | 0 |
| **mEA** |  |  |  |  |  |  |  |  |
| 15 | 70 | 35 | 1,000 | 11 | 66 | 23 | 0 | 0 |
| 16 | 120 | 60 | 400 | 20 | 44 | 36 | 0 | 0 |
| 17 | 90 | 45 | 250 | 2 | 51 | 42 | 5 | 0 |
| 18 | 65 | 32.5 | 450 | 18 | 50 | 28 | 1 | 3 |
| 19 | 100 | 50 | 250 | 12 | 39 | 47 | 1 | 1 |
| ***Median [IQR]*** | 78  [67.5 – 95] | 39.0  [33.8 – 47.5] | 250  [150 – 425] | 12.0  [4.5 – 17.0] | 50  [34.5 – 56.5] | 36  [28.5 – 54.5] | 1.0  [0.0 – 5.0] | 0  [0 – 3.0] |
| **Ctr** |  |  |  |  |  |  |  |  |
| 20 | 70 | 35 | 200 | 0 | 25 | 75 | 0 | 0 |
| 21 | 120 | 60 | 300 | 2 | 26 | 71 | 0 | 0 |
| 22 | 50 | 25 | 100 | 0 | 28 | 73 | 0 | 0 |
| 23 | 60 | 30 | 400 | 0 | 34 | 66 | 0 | 0 |
| 24 | 60 | 30 | 250 | 8 | 44 | 47 | 0 | 0 |
| 25 | 90 | 45 | 300 | 4 | 32 | 64 | 0 | 0 |
| 26 | 80 | 40 | 200 | 5 | 46 | 49 | 0 | 0 |
| 27 | 70 | 35 | 400 | 1 | 25 | 74 | 0 | 0 |
| 28 | 40 | 16 | 200 | 5 | 29 | 66 | 0 | 0 |
| 29 | 120 | 60 | 300 | 9 | 34 | 57 | 0 | 0 |
| 30 | 88 | 44 | 250 | 4 | 40 | 55 | 0 | 1 |

**Supplemental Table 5. Individual horse normalized cytokine result.** Cytokine concentrations normalized to BALF volume returned of IFN-ɣ, CXCL-8, and TNF-α measured via multiplex bead immunoassay in healthy (n=11), mild/moderate asthma (n = 5), and severe asthma (n = 14). Data is represented at the median [IQR] for each group. sEA= Severe Asthma, mEA= Mild/Moderate Asthma, Ctr= Healthy.

| **Horse** | **IFNɣ**  **([pg/mL / mL] * 100)** | **CXCL8**  **([pg/mL / mL] * 100)** | **TNFα**  **([pg/mL / mL] * 100)** |
| --- | --- | --- | --- |
| **sEA** |  |  |  |
| 1 | 353.22 | 202.87 | 397.13 |
| 2 | 51.11 | 27.19 | 7.94 |
| 3 | 817.73 | 70.58 | 92.96 |
| 4 | 40.54 | 40.37 | 2.75 |
| 5 | 87.83 | 31.21 | 5.96 |
| 6 | 33.47 | 25.23 | 5.65 |
| 7 | 811.53 | 204.79 | 107.63 |
| 8 | 189.07 | 69.97 | 85.73 |
| 9 | 126.76 | 34.43 | 30.91 |
| 10 | 41.24 | 34.46 | 21.49 |
| 11 | 97.18 | 42.15 | 13.25 |
| 12 | 82 | 32.57 | 23.38 |
| 13 | 45.83 | 21.52 | 1.74 |
| 14 | 26.02 | 23.02 | 10.60 |
| **Median [IQR]** | 84.92  [41.07 – 230.1] | 34.44  [26.7 – 70.12] | 17.37  [5.88 – 87.54] |
| **mEA** |  |  |  |
| 15 | 41.24 | 35.06 | 10.21 |
| 16 | 17.57 | 12.01 | 4.52 |
| 17 | 54.67 | 13.19 | 4.9 |
| 18 | 285.55 | 68.94 | 10.82 |
| 19 | 106.07 | 6.42 | 8.23 |
| **Median [IQR]** | 54.67  [29.4 – 195.3] | 13.19  [9.21 – 52.0] | 8.23  [4.71 – 10.51] |
| **Ctr** |  |  |  |
| 20 | 30.11 | 2.29 | 1.14 |
| 21 | 17.57 | 1.33 | 0.67 |
| 22 | 42.16 | 2.52 | 1.6 |
| 23 | 48.12 | 20.05 | 3.8 |
| 24 | 35.13 | 10.7 | 7.35 |
| 25 | 23.42 | 10.62 | 2.22 |
| 26 | 26.35 | 1.58 | 1 |
| 27 | 31.49 | 9.69 | 5.93 |
| 28 | 52.7 | 3.15 | 2 |
| 29 | 17.57 | 7.4 | 3.03 |
| 30 | 23.95 | 5.16 | 3.52 |
| **Median [IQR]** | 30.11  [23.42 – 42.16] | 5.16  [2.29 – 10.62] | 2.22  [1.14 – 3.8] |

**Supplemental Table 6. Individual horse raw cytokine result.** Cytokine concentrations of IL-5, IL-17A, IL-2, IL-4, IFN-ɣ, CXCL-8, and TNF-α measured via multiplex bead immunoassay in healthy (n=11), mild/moderate asthma (n = 5), and severe asthma (n = 14). Numbers in italics indicate this value was the software assigned lowest detectable concentration for the individual analyte. Data is represented at the median [IQR] for each group. sEA= Severe Asthma, mEA= Mild/Moderate Asthma, Ctr= Healthy. Numbers in italics were the software assigned lowest detectable concentration.

| **Horse** | **IL-5**  **pg/mL** | **IL-17A**  **pg/mL** | **IL-2**  **pg/mL** | **IL-4**  **pg/mL** | **IFNɣ**  **pg/mL** | **CXCL8**  **pg/mL** | **TNFα**  **pg/mL** |
| --- | --- | --- | --- | --- | --- | --- | --- |
| **sEA** |  |  |  |  |  |  |  |
| 1 | *0.5* | *0.49* | *0.44* | *16.35* | 81.24 | 46.66 | 91.34 |
| 2 | 0.92 | *0.49* | 0.46 | *16.35* | 31.69 | 16.86 | 4.92 |
| 3 | 28.38 | *0.49* | 20.66 | 120.22 | 981.27 | 84.69 | 111.55 |
| 4 | *0.5* | *0.49* | *0.44* | *16.35* | *21.08* | 20.99 | 1.43 |
| 5 | *0.5* | *0.49* | 0.55 | *16.35* | *21.08* | 7.49 | 1.43 |
| 6 | 2.2 | *0.49* | 0.46 | *16.35* | 26.11 | 19.68 | 4.41 |
| 7 | *0.5* | *0.49* | 0.46 | *16.35* | 154.19 | 38.91 | 20.45 |
| 8 | 1.6 | *0.49* | 0.46 | *16.35* | 56.72 | 20.99 | 25.72 |
| 9 | 1.46 | *0.49* | 2.48 | *16.35* | 73.52 | 19.97 | 17.93 |
| 10 | 1.05 | *0.49* | *0.44* | *16.35* | 28.87 | 24.12 | 15.04 |
| 11 | *0.5* | *0.49* | *0.44* | *16.35* | 38.87 | 16.86 | 5.3 |
| 12 | 3.02 | *0.49* | 0.86 | 19.98 | 49.2 | 19.54 | 14.03 |
| 13 | *0.5* | *0.49* | *0.44* | *16.35* | *21.08* | 9.9 | *0.8* |
| 14 | 1.9 | *0.49* | *0.44* | *16.35* | *21.08* | 18.65 | 8.59 |
| **mEA** |  |  |  |  |  |  |  |
| 15 | 0.8 | *0.49* | *0.44* | *16.35* | 28.87 | 24.54 | 7.15 |
| 16 | 0.63 | *0.49* | *0.44* | *16.35* | *21.08* | 14.41 | 5.42 |
| 17 | 6.44 | *0.49* | 0.91 | 31.96 | 49.2 | 11.87 | 4.41 |
| 18 | 11.24 | *0.49* | 2.54 | 105.48 | 184.96 | 44.81 | 7.03 |
| 19 | *0.5* | *0.49* | *0.44* | *16.35* | 106.07 | 6.42 | 8.23 |
| **Ctr** |  |  |  |  |  |  |  |
| 20 | *0.5* | *0.49* | *0.44* | *16.35* | *21.08* | 1.6 | *0.8* |
| 21 | *0.5* | *0.49* | *0.44* | *16.35* | *21.08* | 1.6 | *0.8* |
| 22 | 0.99 | *0.49* | *0.44* | *16.35* | *21.08* | *1.26* | *0.8* |
| 23 | 3.02 | *0.49* | *0.44* | *16.35* | 28.87 | 12.03 | 2.28 |
| 24 | *0.5* | *0.49* | *0.44* | *16.35* | *21.08* | 6.42 | 4.41 |
| 25 | 0.86 | *0.49* | *0.44* | *16.35* | *21.08* | 9.56 | 2 |
| 26 | *0.5* | *0.49* | *0.44* | *16.35* | *21.08* | *1.26* | *0.8* |
| 27 | *0.5* | *0.49* | *0.44* | *16.35* | 22.04 | 6.78 | 4.15 |
| 28 | *0.5* | *0.49* | *0.44* | *16.35* | *21.08* | *1.26* | *0.8* |
| 29 | *0.5* | *0.49* | *0.44* | *16.35* | *21.08* | 8.88 | 3.63 |
| 30 | 3.02 | *0.49* | *0.44* | 28.99 | *21.08* | 4.54 | 3.1 |

**Supplemental Table 7.** **Percent cytokine recovered.**

| **Analyte** | **Expected (pg/mL)** | **Observed (pg/mL)** | **% Recovered** |
| --- | --- | --- | --- |
| **IL-5** | 3.66 | 4.49 | 122.68 |
|  | 14.65 | 15.06 | 102.80 |
|  | 58.59 | 62.36 | 106.43 |
|  | 234.38 | 238.25 | 101.65 |
|  | 937.5 | 866.24 | 92.40 |
|  | 3750 | 3336.18 | 88.96 |
| **IL-17A** | 3.66 | 2.37 | 64.75 |
|  | 14.65 | 5.65 | 59.04 |
|  | 58.59 | 37.58 | 64.14 |
|  | 234.38 | 143.81 | 61.36 |
|  | 937.5 | 543.24 | 57.95 |
|  | 3750 | 2023 | 53.95 |
| **IL-2** | 3.66 | 3.84 | 104.92 |
|  | 14.65 | 16.67 | 113.79 |
|  | 58.59 | 67.74 | 115.62 |
|  | 234.38 | 254.92 | 108.76 |
|  | 937.5 | 1058.77 | 112.94 |
|  | 3750 | 3955.77 | 105.49 |
| **IL-4** | 73.24 | 130.78 | 178.56 |
|  | 292.97 | 435.57 | 148.67 |
|  | 1172 | 1469 | 125.34 |
|  | 4688 | 5494 | 117.19 |
|  | 18750 | 17736 | 94.59 |
|  | 75000 | 71106 | 94.81 |
| **IFNɣ** | 122.07 | 133.4 | 109.28 |
|  | 488.28 | 513.67 | 105.2 |
|  | 1953 | 1942.76 | 99.48 |
|  | 7812 | 7485.76 | 95.82 |
|  | 31250 | 27647.76 | 88.47 |
|  | 125000 | 103586.76 | 82.87 |
| **CXCL8/IL-8** | 14.65 | 18.78 | 128.19 |
|  | 58.89 | 54.56 | 93.12 |
|  | 234.38 | 201.51 | 85.98 |
|  | 937.5 | 844.48 | 90.08 |
|  | 3750 | 3449 | 91.97 |
|  | 15000 | 8979 | 59.86 |
| **TNFα** | 0.98 | 2.82 | 287.75 |
|  | 3.91 | 4.99 | 127.62 |
|  | 15.62 | 18.54 | 118.69 |
|  | 62.5 | 66.74 | 106.78 |
|  | 250 | 270.67 | 108.27 |
|  | 1000 | 1056 | 105.6 |
